# Protocol to dissociate, process, and analyze the human lung tissue using single-cell RNA-seq

**DOI:** 10.1016/j.xpro.2022.101776

**Published:** 2022-10-21

**Authors:** Álvaro Quintanal-Villalonga, Joseph M. Chan, Ignas Masilionis, Vianne Ran Gao, Yubin Xie, Viola Allaj, Andrew Chow, John T. Poirier, Dana Pe’er, Charles M. Rudin, Linas Mazutis

**Affiliations:** 1 Department of Medicine, Thoracic Oncology Service, Memorial Sloan Kettering Cancer Center, New York, NY, USA; 2 Program for Computational and Systems Biology, Sloan Kettering Institute, Memorial Sloan Kettering Cancer Center, New York, NY, USA; 3 Perlmutter Cancer Center, New York University Langone Health, New York, NY, USA; 4 Parker Institute for Cancer Immunotherapy, Memorial Sloan Kettering Cancer Center, New York, NY, USA; 5 Institute of Biotechnology, Life Sciences Centre, Vilnius University, Vilnius, Lithuania

**Keywords:** Cancer, Cell isolation, Flow cytometry/Mass cytometry, Molecular biology, RNAseq, Sequence analysis, Sequencing, Single cell

## Abstract

We report a protocol for obtaining high-quality single-cell transcriptomics data from human lung biospecimens acquired from core needle biopsies, fine-needle aspirates, surgical resection, and pleural effusions. The protocol relies upon the brief mechanical and enzymatic disruption of tissue, enrichment of live cells by fluorescence-activated cell sorting (FACS), and droplet-based single-cell RNA sequencing (scRNA-seq). The protocol also details a procedure for analyzing the scRNA-seq data.

For complete details on the use and execution of this protocol, please refer to Chan et al. (2021).

## Highlights

• Preparation of diverse human lung biospecimens for scRNA-seq
• Enrichment for live cells by FACS
• Applicable to different scRNA-seq platforms
• Computational workflow provides critical guideline for step-by-step data analysis

## Abstract (editor highlights)

Publisher’s note: Undertaking any experimental protocol requires adherence to local institutional guidelines for laboratory safety and ethics.

## Graphical abstract

**Figure:**
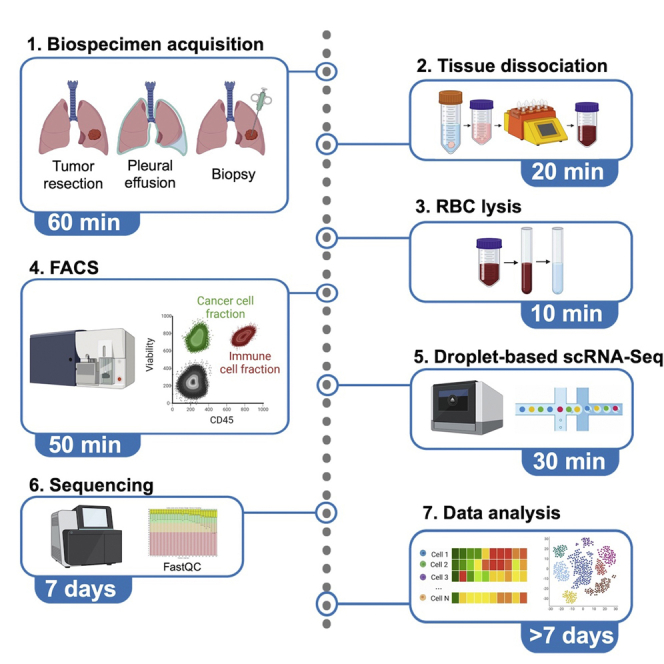

## Before you begin

Follow institutional guidelines to obtain a permit for working with patient derived material. Patient consent must be acquired for all human samples. Follow all safety precautions governing work with biohazardous materials: wear lab coats, gloves, and safety glasses at all times. Properly dispose the biological and chemical materials, decontaminate work surfaces. Use pipette tips with aerosol barriers that are sterile and free from DNases and RNases.

A critical factor to maximize viability and ensure quality of data derived from this procedure is to limit ischemic time, understood as the time from specimen extraction from the patient until dissociation starts, to under an hour. Longer ischemic times may lead to increased cell death and mRNA degradation by ribonucleases present in the tissue. Surgical samples should be placed in phosphate buffered saline [pH 7.4], or cell culture media (e.g., RPMI-1640, DMEM) after resection, and transported to the lab on ice.

### Institutional permissions

All experiments performed using the methodologies herein described on clinical specimens were approved by an Institutional Review Board. All patients from whom biospecimens were obtained provided informed consent through an Institutional Review Board-approved biospecimen collection and analysis protocol.

## Key resources table

**Table:** 

REAGENT or RESOURCE	SOURCE	IDENTIFIER
**Antibodies**		

PE anti-human CD45 antibody (Working dilution: 3 μL/100 μL)	BioLegend	#368510 RRID: AB_2566370

**Chemicals, peptides, and recombinant proteins**		

RPMI 1640 [+] L-glutamine, 25 mM HEPES	Corning	#10-041-CV
DPBS (Dulbeccos Phosphate Buffered Saline) 1× [-] calcium magnesium	Corning	#21-031-CV
Fetal Bovine Serum	Gemini Bio-Products	#900-108
ACK Lysing Buffer (1×) (red blood cell lysis buffer)	Lonza	#10-548E
Human TruStain FcX	BioLegend	#422302
Calcein AM	BioLegend	#425201
DAPI (4,6-Diamidino-2-Phenylindole Dihydrochloride)	Invitrogen	#D1306
Ficoll Paque PLUS	GE Healthcare	#17144003-500 mL
0.4% (w/v) Trypan Blue Solution	Thermo Fisher Scientific	#15250061
Ethanol, Pure (anhydrous)	Sigma	#E7023-500ML
Nuclease-free water	Thermo Fisher Scientific	# AM9937
DNA/RNA Free Reagent Spray	Argos Technologies	# UX-04397-24
Tris-HCl [pH 8.5]	Avantor	# MB-027-1000

**Critical commercial assays**		

Human Tumor Dissociation Kit	Miltenyi Biotec Inc.	#130-095-929
Chromium Single Cell 3′ GEM, Library & Gel Bead Kit v3, 16 rxns	10× Genomics	#1000075
Chromium i7 Multiplex Kit, 96 rxns	10× Genomics	#120262
NovaSeq 6000 S2 Reagent Kit v1.5 (100 cycles)	Illumina	# 20028316
SPRIselect Reagent Kit	Beckman Coulter	#B23318

**Deposited data**		

scRNA-seq and MIBI data	HTAN Data Portal; CZI	https://data.humantumoratlas.org/ https://cellxgene.cziscience.com/collections/62e8f058-9c37-48bc-9200-e767f318a8ec

**Software and algorithms**		

SEQC	Azizi et al., 2018	https://github.com/dpeerlab/seqc
CB2	Ni et al., 2020	https://github.com/zijianni/scCB2
DoubletDetection	Gayoso et al. (2019)	https://github.com/dpeerlab/doubletdetection
scanpy (suite of single-cell algorithms, including UMAP, tSNE, score_genes, among others)	Wolf et al., 2018	https://scanpy.readthedocs.io/en/stable/#
PhenoGraph (includes clustering and Markov absorption modeling)	Haghverdi et al., 2018	https://github.com/dpeerlab/phenograph
fastMNN (through the batchelor package)	Levine et al., 2015	https://github.com/LTLA/batchelor/blob/master/R/fastMNN.R
MAGIC and knnDREMI	Dijk et al. (2018)	https://github.com/dpeerlab/magic
MAST	Finak et al., 2015	https://github.com/RGLab/MAST
Limma	Ritchie et al. (2015)	https://bioconductor.org/packages/release/bioc/html/limma.html
fGSEA	Korotkevich et al. (2021)	https://bioconductor.org/packages/release/bioc/html/fgsea.html
Ambient RNA detection	Smillie et al., 2019	https://github.com/cssmillie/ulcerative_colitis
DirichletReg	Maier (2021)	https://cran.r-project.org/web/packages/DirichletReg/index.html
Cellphonedb	Efremova et al. (2020)	https://github.com/Teichlab/cellphonedb
Survival	Therneau and Grambsch (2000)	https://cran.r-project.org/web/packages/survival/index.html
Non-negative matrix factorization in Scikit-learn v. 20.0	Pedregosa et al. (2011)	https://scikit-learn.org/stable/
Vectra Imaging Processing Pipeline	N/A	https://github.com/dpeerlab/Vectra_Imaging_pipeline
MaskRCNN_cell (segmentation for Vectra image)	N/A	https://github.com/dpeerlab/MaskRCNN_cell
ARK-analysis (MIBI analysis)	N/A	https://github.com/angelolab/ark-analysis
Mesmer	Greenwald et al. (2021)	https://github.com/vanvalenlab/deepcell-tf
Squidpy	Palla et al. (2022)	https://github.com/theislab/squidpy/

**Other**		

GentleMACS Octo Dissociator with Heaters	Miltenyi Biotec	#130-096-427
Chromium Controller	10× Genomics	#PN-120223
Thermal Cycler, C1000 Touch with 96-Deep Well Reaction Module, or alternative	Bio-Rad	#1851197
Aspiration System (e.g., Vacusafe, or alternative)	INTEGRA Biosciences	#158300
2100 Bioanalyzer Laptop Bundle	Agilent	#G2943CA
Qubit 4.0 Flourometer	Thermo Fisher Scientific	#Q33226
Countess II	Thermo Fisher Scientific	#AMQAX1000
Swinging bucket cooling centrifuge (e.g., Sorvall Legend X1 Centrifuge Series), or alternative	Fisher Scientific	# 75004220
Vortex Genie-2, or alternative	Scientific Industries	#SI-0236
BD FACS Aria II, or alternative	BD Biosciences	N/A
Inverted Phase Contrast Microscope (Nikon Eclipse TS100, or similar)	Nikon	#14003
GentleMACS C tube	Miltenyi Biotec	#130-093-237
MACS SmartStrainers (70 μm)	Miltenyi Biotec	#130-098-462
Chromium Chip B Single Cell Kit, 48 rxns	10× Genomics	#1000073
Corning Falcon Test Tube with 35 μm Cell Strainer Snap Cap	Corning	#352235
High Sensitivity DNA Kit	Agilent	#5067-4626
Qubit dsDNA HS Assay Kit	Thermo Fisher Scientific	#Q32854
Countess™ Cell Counting Chamber Slides	Thermo Fisher Scientific	#C10228
SepMate-50 (IVD) 100 Tubes	STEMCELL Technologies	#85450
PCR Tubes 0.2 mL 8-tube strips	Eppendorf	# 951010022
1.5 mL Protein LoBind Tubes	Eppendorf	# 0030108442
15 mL tubes	Thermo Scientific	#339650
50 mL tubes	CELLTREAT	#229421
Cell culture dish – 100 mm diameter	Corning	#430167
Glass Pasteur pipettes	DWK Life Sciences (Kimble)	#63B92
Razor blades	Thermo Fisher Scientific	#12-640
Specimen Forceps, Serrated, Straight, Length=114 mm	VWR	#82027-440
Pipettes P2, P20, P100, P200 and P1000 (e.g., Eppendorf, Rainin, Gillson, etc) and corresponding pipette tips.	Rainin	#30389240 #30389213 #30389226

## Materials and equipment

**Table:** Cell Resuspension buffer

Reagent	Final concentration	Amount
1× PBS	N/A	97.5 mL
FBS	2.5% (v/v)	2.5 mL
**Total**	**–**	**100 mL**

Store at 4°C for 1 week.

**Table:** RPMI-FBS buffer

Reagent	Final concentration	Amount
RPMI-1640 medium	N/A	9.75 mL
FBS	2.5% (v/v)	0.25 mL
**Total**	**–**	**10 mL**

Store at 4°C for up to 1 week.

**Table:** Cell Staining Mix for live-cell enrichment by FACS

Reagent	Final concentration	Amount (for staining 10^6^ cells)
Cell Resuspension Buffer	N/A	99.5 μL
Calcein AM (1 mM)	5 μM	0.5 μL
**Total**	**–**	**100 μL**

Prepare fresh on a day of experiment.

**Table:** Cell Staining Mix for CD45-negative live-cell enrichment by FACS

Reagent	Final concentration	Amount (for staining 10^6^ cells)
Cell Resuspension Buffer	N/A	91.5 μL
Human TruStain FcX	N/A	5 μL
PE anti-human CD45 antibody	N/A	3 μL
Calcein AM (1 mM)	5 μM	0.5 μL
**Total**	**–**	**100 μL**

Prepare fresh on a day of experiment.

## Step-by-step method details

**Figure 1:**
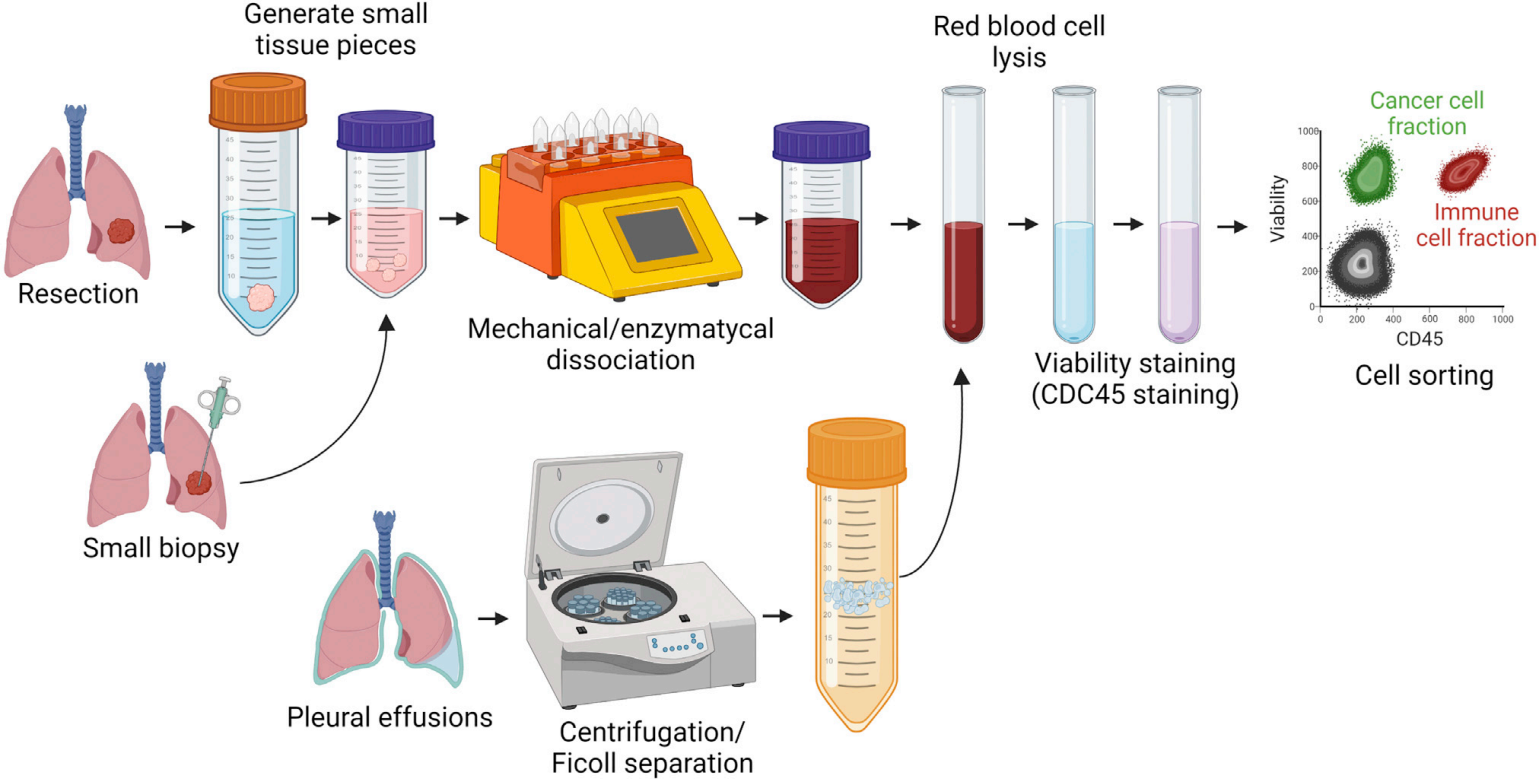
Overview of the protocol for preparing of human lung biospecimens for droplet-based scRNA-seq The main steps of the protocol involve mechanical & enzymatic dissociation of lung tumor, red blood cell lysis, staining of cells with live dye (Calcein AM) and fluorescently-labeled anti-human CD45 antibody, and fluorescence-activated cell sorting (FACS) of live-cells.

The protocol below describes the specific steps for processing human lung tumor specimens, which are schematically indicated in Figure 1. The specimen is dissociated using a combination of hydrolytic enzymes and mechanical sharing followed by FACS enrichment for live-cells to obtain a single-cell suspension applicable for scRNA-seq. The processing protocol will be specific to the specimen type. For typical resections (>100 mg), proceed to Part A. Surgical specimen dissociation section (Part A, steps 1–13). For core needle biopsies (at least one core, typically 10–100 mg) or fine needle aspirates (typically 10–50 mg), proceed to Part B. Small size specimen dissociation (core needle biopsies, fine needle aspirations) section (Part B, steps 14–23). For pleural effusion samples, proceed to Part C. Pleural effusion processing section (Part C, steps 24–34).

### Part A. Surgical specimen dissociation

**Timing: 1 h**

To achieve a high quality scRNA-seq data it is critical to ensure speedy sample processing. Dissociation of clinical samples should begin immediately, within 1 to 2-h window after specimen retrieval from the patient. Delaying the dissociation is likely to lead to increased cell death and mRNA degradation. Throughout the protocol all centrifugations should be performed in a swinging bucket centrifuge unless specified otherwise.

1. Place a surgical specimen in a tube filled with 5–20 mL of 1× PBS and place on ice. The volume of 1× PBS can be adjusted according to the size of tissue. Ensure that biospecimen is completely submerged.

***Note:*** The type of tube at this step is not critical at this step. We typically use 50 mL Falcon tubes.

**Figure 2:**
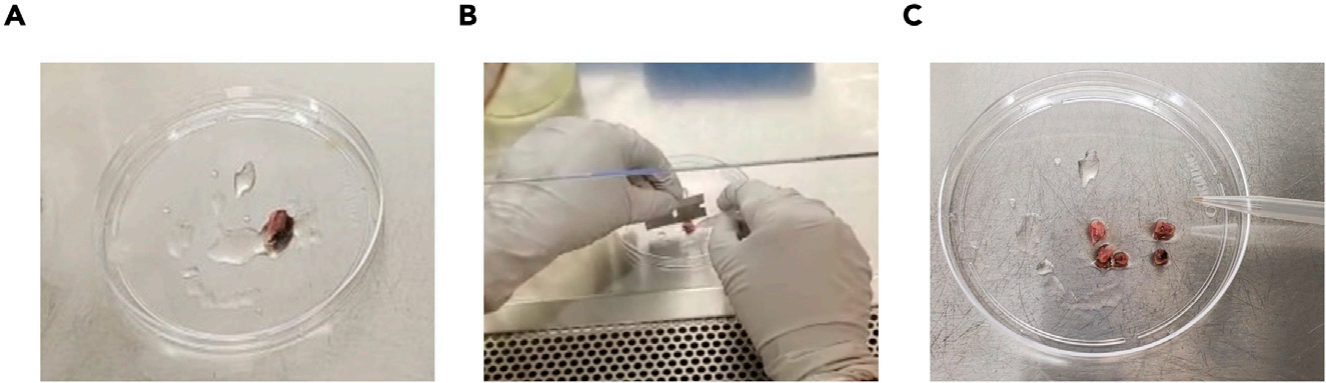
Preparation of human lung biospecimen for dissociation (A) Tumor piece placed in a Petri dish. (B) Cutting procedure. The tumor piece can be held with tweezers or a pipette tip with one hand, while using the other hand to cut the tissue with the razor blade into smaller pieces. (C) Tumor cut into ∼5 mm^3^ pieces before dissociation.
5. Using the tweezers transfer the tissue pieces to the gentleMACS C Tube containing the enzyme mix prepared at step #3.
6. Place the gentleMACS C-Tube in the gentleMACS Octo Dissociator with Heaters, and run the default program “37C_H_TDK3” for 15 min. Inspect the quality of dissociation by eye.

***Note:*** If no tissue chunks are visible to the naked eye proceed to next step, if the suspension contains pieces of tissue run the program for additional 10 min. The tissue dissociation step involves mechanical, enzymatic and temperature stresses to the cells, therefore minimizing the dissociation time is important. Extended dissociation may damage fragile cells, reduce cell recovery and RNA quality.

2. While biospecimen is kept on ice, in a separate 1.5 mL laboratory tube prepare the enzyme mix comprising 250 μL of Enzyme H, 110 μL of Enzyme R and 40 μL μL of Enzyme A provided in the Human Tumor Dissociation Kit (Miltenyi Biotec).
3. Transfer enzyme mix from step #2 into the gentleMACS C-Tube (Miltenyi Biotec) supplemented with 7.5 mL of serum-free cell culture medium (e.g., RPMI-1640).
4. Aspirate the buffer in which the biospecimen is submerged and transfer the tissue to a 15 mm Petri dish. While holding it with tweezers, cut the specimen into ∼5 mm^3^ pieces using a sharp razor (Figure 2).

7. Pass the dissociated cells through a 70 μm cell strainer (e.g., MACS SmartStrainer) placed in a 50 mL conical tube.
8. Gently rinse the 70 μm cell strainer with 20–25 mL of Cell Resuspension buffer.
9. Split the flow-through suspension into two 15 mL canonical tubes and spin at 800 × *g* for 3 min at 20°C–25°C.
10. Aspirate the supernatant carefully with a glass Pasteur pipette coupled to a vacuum source without disturbing the cell pellet. Leaving ∼50–100 μL of supernatant on top of the pellet.

***Note:*** Follow institutional guidelines to dispose biohazard. For example, the supernatant might be disposed into a 1% bleach solution.

11. Red Blood Cell Lysis.
  a. Gently resuspend the cell pellet in 1–2 mL of Red Blood Cell Lysis Solution (ACK buffer, Lonza) by slowly pipetting up and down 2–3 times using 1 mL pipette. Incubate for 2 min at 20°C–25°C.
    ***Note:*** This step should be performed even if red blood cells are not visually evident.
  b. Dilute the ACK buffer by adding 20 mL of Cell Resuspension buffer.
  c. Split the suspension into two 15 mL tubes, and centrifuge at 800 × *g* at 20°C–25°C for 3 min.
  d. Aspirate the supernatant without disturbing the cell pellet. At this point, the cell pellet should be whitish or yellowish.
    ***Note:*** If cell pellet at this step appears light red, repeat RBC lysis steps.

12. Gently resuspend the cell pellet in 3–4 mL of refrigerated Cell Resuspension buffer, transfer onto a 35 μm Cell Strainer Snap Cap and let the fluid pass through the filter by gravity.
13. Place the collected flow through fraction onto the ice bucket and proceed to “Part D. Cell staining with live-dye and anti-CD45 antibody” section.

### Part B. Small size specimen dissociation (core needle biopsies, fine needle aspirations)

**Timing: 30 min**

The dissociation yield of smaller size specimens (approximately 2 × 2 × 10 mm^3^) tends to be higher when reduced volumes of buffers are used, and when tissue dissociation is conducted for a shorter period of time. Given small size of the specimens, an extra care needs to be taken at each step to minimize the cell loss.

14. Use Human Tumor Dissociation Kit (Miltenyi Biotec) to prepare the enzyme mix comprising 85 μL of Enzyme H, 40 μL of Enzyme R and 15 μL μL of Enzyme A.
15. Transfer enzyme mix from step #13 into the gentleMACS C-Tube (Miltenyi Biotec) supplemented with 2.5 mL of serum-free cell culture medium (e.g., RPMI-1640).
16. Centrifuge the tube having a core biopsy piece(s) at 800 × *g* for 2 min at 20°C–25°C. Discard the supernatant and transfer the tissue pieces to the GentleMACS C-Tube containing the enzyme mix.
17. Place the GentleMACS C-Tube in the GentleMACS Octo Dissociator with Heaters (Miltenyi), and run default program “37C_H_TDK3” for 15 min.
18. Verify the dissociation by visually inspecting the solution. If the tissue is not completely dissociated, continue running the program for additional 5 min.

***Note:*** The dissociation step involves mechanical, enzymatic and temperature stresses to the cells, therefore minimizing the dissociation time is important. Extended dissociation may damage fragile cells and reduce RNA quality. The tissue is considered dissociated when the solution becomes cloudy and no tissue chunks are visible to the naked eye.

19. Apply the cell suspension obtained in step #16 onto 35 μm Cell Strainer Snap Cap placed on the Test Tube (Corning) and let the suspension pass the strainer by gravity.
20. Rinse the GentleMACS C Tube with 1.5 mL of Cell Resuspension Buffer (equilibrated at 20°C–25°C) to collect the left-over cells, transfer the suspension onto the same Cell Strainer Snap Cap and collect the flow through fraction into the same Test Tube.
21. Spin the Test Tube at 800 × *g* for 2 min at 20°C–25°C. Carefully aspirate the supernatant using a glass Pasteur pipette coupled to a vacuum source without disturbing the cell pellet.

***Note:*** To prevent disturbance of cell pellet leave ∼30–50 μL of supernatant on top of the cell pellet.

22. Red Blood Cell Lysis.
  a. Add 200 μL of Red Blood Cell Lysis Solution (ACK buffer, Lonza) on a cell pellet and gently resuspend by slowly pipetting up and down 2–3 times. Incubate for 2 min at 20°C–25°C.
    ***Note:*** Always include RBC lysis even if blood cells are not visually evident in the sample.
  b. Dilute the ACK buffer by adding 2 mL of Cell Resuspension buffer and centrifuge at 800 × *g* for 2 min at 20°C–25°C.
  c. Aspirate the supernatant without disturbing the cell pellet, leaving ∼30 μL of supernatant on top of the cell pellet. At this point, the cell pellet should be whitish or yellowish.
    ***Note:*** If cell pellet at this step appears light red, repeat RBC lysis steps.

23. Place the tube with cell suspension onto the ice bucket and proceed to “Part D. Cell staining with live-dye and anti-CD45 antibody” section.

### Part C. Pleural effusion processing

**Timing: 30 min**

Malignant pleural effusions that form in lung cancer patients derive from fluid accumulation in the pleural cavity. Typically, the volume of pleural effusion samples varies from 50 to 1,000 mL. Macrophages, T-cells and tumor cells can be found in the pleural effusions at concentrations ranging from a dozen to thousands of cells per 1 mL, however, such concentrations are too low for direct droplet-based scRNA-seq, and therefore concentrating the cells down to ∼1,000 cells/μL is required.

24. To concentrate the cells, transfer the pleural effusion fluid into a required number of conical 50 mL tubes and centrifuge at 500 × *g* at 20°C–25°C for 10 min.
25. Carefully remove the supernatant leaving ∼50 μL of supernatant on top of the cell pellet.
26. Carefully disperse the cell pellet in the supernatant that was left in the tube. Repeat it for each tube.
27. Combine all cell suspensions in 15–20 mL of Cell Resuspension buffer.

***Note:*** The resuspension volume depends on the size of the pellets obtained. 20 mL of cell resuspension buffer might be used per 250 mL of initial pleural effusion volume.

28. To purify the mononuclear cells, prepare SepMate-50 (IVD) tubes (STEMCELL Technologies) by loading ∼15 mL of Ficoll-Paque PLUS (GE Healthcare) into tube’s separating hole until the fluid level is just above the dividing plastic.

***Note:*** One SepMate-50 (IVD) tube is required for up to 20 mL of concentrated cell suspension from step #22.

29. Slowly layer 20 mL of concentrated cell suspension onto the Ficoll-Paque PLUS in the SepMate-50 (IVD) tube.

**CRITICAL:** When layering the sample, minimize the mixing of the Ficoll-Paque PLUS solution and the cell suspension, as that could compromise adequate separation.

30. Centrifuge SepMate-50 (IVD) tube at 1,200 × *g* for 10 min at 20°C–25°C, with the centrifuge accelerator and break turned off.
31. Remove 15 mL of upper fluid layer from each SepMate-50 (IVD) tube into a 50 mL Falcon tube. For the remaining ∼5 mL above the dividing plastic, gently pipette up and down to disperse and collect the residue residing above the dividing plastic.
32. Centrifuge at 800 × *g* at 20°C–25°C for 3 min and discard the supernatant leaving ∼50 μL of supernatant on top of the cell pellet.
33. Red Blood Cell Lysis.
  a. Add 200 μL of Red Blood Cell Lysis Solution (ACK buffer, Lonza) on a cell pellet and gently resuspend by slowly pipetting up and down 2–3 times. Incubate for 2 min at 20°C–25°C.
    ***Note:*** Always include RBC lysis even if blood cells are not visually evident in the sample.
  b. Dilute the ACK buffer by adding 2 mL of Cell Resuspension buffer.
  c. Centrifuge at 800 × *g* for 2 min at 20°C–25°C. Aspirate the supernatant without disturbing the cell pellet, leaving ∼30 μL of supernatant on top of the cell pellet. At this point, the cell pellet should be whitish or yellowish.
    ***Note:*** If cell pellet at this step appears light red, repeat RBC lysis steps.

34. Proceed to “Part D. Cell staining with live-dye and anti-CD45 antibody” section.

### Part D. Cell staining with live-dye and anti-CD45 antibody

**Timing: 25 min**

Cell staining with Calcein AM dye enables live cell identification. When this chemical dye is used in combination with fluorescently-labelled anti-CD45 antibody staining, the live-immune cells (CD45 positive fraction) and live-nonimmune cells (CD45 negative fraction) can be efficiently differentiated and selectively enriched. Staining cells against CD45 is particularly advantageous when tumor cells (CD45 negative) constitute a relatively small fraction (0.1%–10%) of the total tumor mass (e.g., in lung adenocarcinomas). The negative selection strategy (depletion of CD45 positive cells) provides an option to enrich cancer cells in the final cell suspension. Staining with anti-CD45 antibody is unnecessary when tumor cell enrichment is not required. However, sorting for live-cells is highly recommended for ensuring the high-quality of scRNA-seq data.

35. Centrifuge the cells in FACS tube at 800 × *g* for 3 min at 20°C–25°C and aspirate supernatant carefully without disturbing the cell pellet. Leave 50 μL of supernatant on top of the cell pellet.
36. During centrifugation, prepare either Cell Staining Mix for live-cells, or Cell Staining Mix for CD45-negative cells (see materials and equipment section for Cell Staining Mix composition).
37. Combine the 50 μL cell suspension from step-32 and 100 μL Cell Staining Mix per roughly 10^6^ cells from step-33. Gently rock the tube or pipette up and down 2–3 times to disperse the cells.
38. Incubate on ice for 15 min.
39. Dilute the Cell Staining Mix in step-34 by adding 2 mL of refrigerated Cell Resuspension Buffer and centrifuge in a swinging bucket centrifuge at 800 × *g* for 3 min.
40. Aspirate supernatant carefully, add 2 mL of RPMI-FBS (RPMI-1640 medium supplemented with 2.5% (v/v) FBS), and centrifuge at 800 × *g* for 3 min.
41. Without further delays proceed to “Part E. FACS: live-cell enrichment” section.

### Part E. FACS: Live-cell enrichment

**Timing: 30 min**

Generally, the cell sorting and enrichment may be performed on any conventional FACS instrument and does not require a special setup. The sorting settings are instrument specific. In this protocol we used BD FACS Aria II instrument equipped with nozzle 100 μm wide and sorting at speed below 6,000 events/second. It is important that the FACS-sorted cells are collected in Protein LoBind tubes, which possess reduced cell adhesion properties and thus increase cell recovery. However, because cell pellet can be easily dislodged easily in these tubes during pipetting, the aspiration of the supernatant after centrifugation should be performed carefully. The addition of 50 μL of RPMI-FBS medium will reduce cell death due to sample desiccation during the first minutes of sorting.

**Figure 3:**
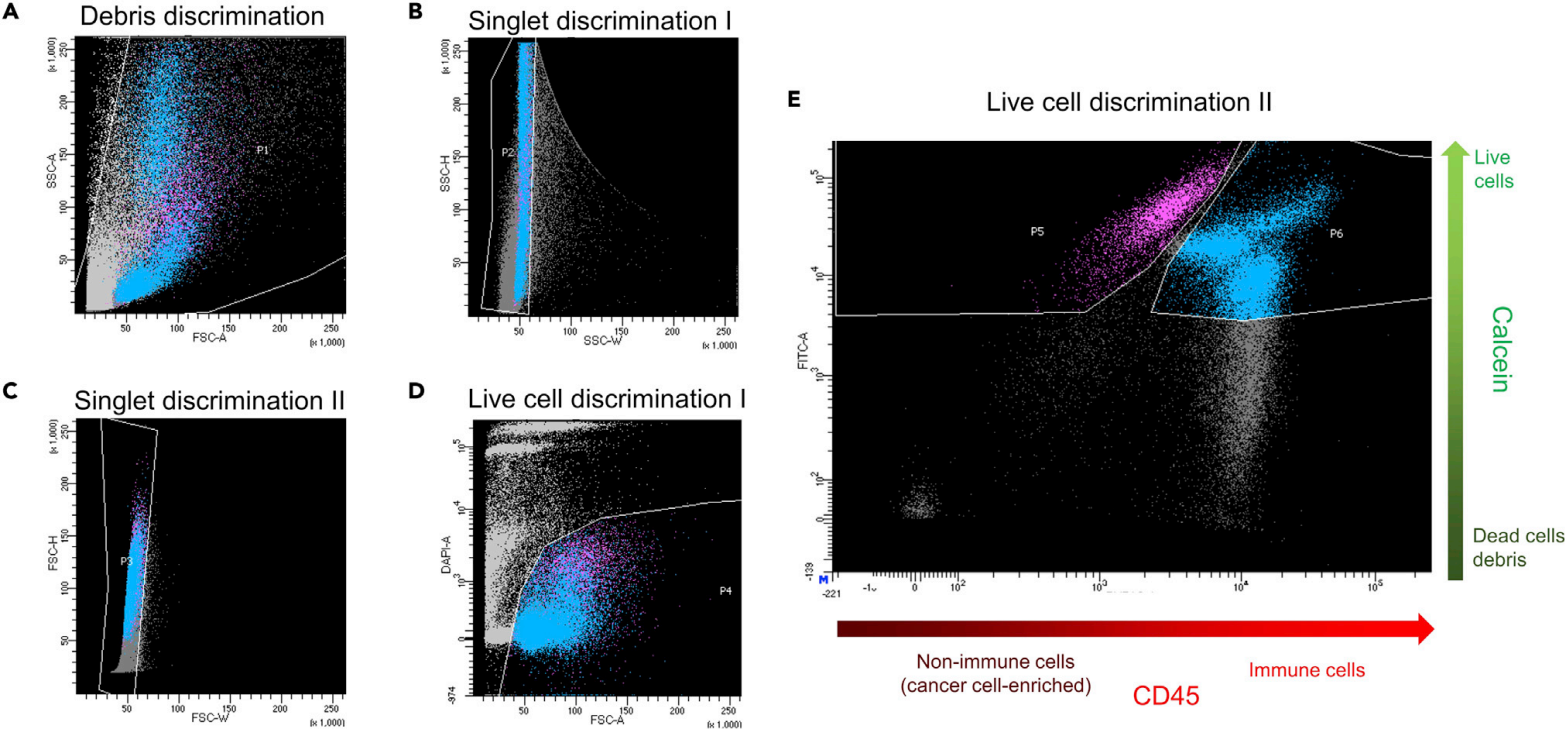
Setting the FACS gates for enrichment of live immune cells (CD45-positive) and live non-immune (CD45-negative) cells (A) The side scatter (SSC-A) and forward scatter (FSC-A) plot can be used to discard debris, however, we recommend to be inclusive in this gating to avoid potential loss of the cells of interest. (B) Side scatter height (SSC-H) and side scatter width (SSC-W) plot. (C) Forward scatter height (FSC-H) and forward scatter width (FSC-W) plot, are used to gate singlets. (D) DAPI and forward scatter plot facilitates identification of live-cells (DAPI negative), but we recommend again to be inclusive, as the complexity of clinical samples sometimes hurdles the clear identification of live cells. (E) FITC (Calcein) and PE (CD45) scatter plot is used to enrich live-cells that are positive for FITC. Immune cells (CD45 positive) and non-immune cells (CD45 negative) should be clearly separated in the PE (CD45) channel. Noteworthy that CD45-positive cells include several clusters in the flow cytometry plot, which mainly correspond to different immune cell types (T-cells, macrophages, etc.).

42. Resuspend the cell pellet in ice-cold 100 μL RPMI-FBS. Add DAPI dye at a final concentration of 0.1–1 μg/mL, and place the tube on ice.
43. Prepare 1.5 mL Protein LoBind Eppendorf tubes with 50 μL of RPMI-FBS that will serve as collection tube for FACS-sorted cells.
44. Follow the instructions of the corresponding FACS instrument to initiate the flow cytometry.
45. Set appropriate sorting gates for capturing the live-cell populations of interest (see Figure 3).
  ***Note:*** The CD45-positive cells will comprise several subpopulations of cells, accounting for the different cell types (T-cells, macrophages, etc.), which will appear as different clusters in the flow cytometry plot.
  a. For enriching the live non-immune (tumor-enriched) cells set the sorting gates negative for DAPI and PE, and positive for Calcein.
  b. For enriching the live immune cells set the sorting gates negative for DAPI and positive for Calcein and PE.
    ***Note:*** If tumor cell enrichment is not required and CD45 staining was not performed, set the sorting gate negative for DAPI and positive for Calcein dyes. This will account for all live-cells.

46. Initiate cell sorting and collect FACS-sorted cells into 1.5 mL Protein LoBind tube filled with 50 μL of RPMI-FBS.

***Note:*** Knowing the exact count of CD45neg and CD45pos events during FACS provides an option for later combining the sorted cells at a desirable ratio and quantity. This might be particularly desirable in the situations when tumor cells constitute only a small fraction of all cells or when sorted CD45neg cells results in numbers insufficient for droplet-based scRNA-seq approach. Therefore, following FACS the sorted CD45 negative cells (representing the non-immune compartment) can be spiked-in with a known quantity of sorted CD45 positive cells (representing the immune compartment) to obtain an adequate cell concentration for droplet-based scRNA-seq.

47. Record the cell count during FACS.
  a. If sorted CD45neg cell count is ≥50.000, proceed to the next step.
  b. If sorted CD45neg cell count ≤50.000, combine the sorted CD45neg with sorted CD45pos cells to obtain at least 50.000 cells in total.
48. Centrifuge cells at 800 × *g* for 4 min at 4°C.
49. Without disturbing the cell pellet, carefully aspirate the supernatant and gently resuspend the cell pellet in a required volume of RPMI-FBS medium to obtain ∼1,500 cells/μL. For example, if FACS count showed 50.000 cells, resuspend the cell pellet in 20–30 μL of RPMI-FBS medium.

***Note:*** In our experience the FACS count often turns out to be higher than the actual number of sorted cells. Furthermore, concentrating cells by centrifugation and subsequent removal of the supernatant, may also lead to undesirable cell losses. Therefore, when resuspending the cells in a final volume it is worth to consider the actual number of the cells in a pellet to be 30%–50% lower than what appears on a FACS instrument.

50. Without further delays proceed to “Part F. Droplet-based single-cell RNA-Seq” section.

### Part F. Droplet-based single-cell RNA-Seq

**Timing: 20–30 min**

The cells enriched with FACS are readily applicable for use with different high-throughput droplet microfluidic platforms (Klein et al., 2015; Macosko et al., 2015; Zheng et al., 2017), however, as cells tend to lose viability over time it may be desirable to choose the platform that offers fast sample processing. In our work (Chan et al., 2021), we used Chromium instrument (10× Genomics) in combination with Single Cell 3′ Reagent Kit (v3) to prepare the barcoded transcriptome libraries. For convenience, refer to Methods S1 which provides manual for using Chromium instrument and reagent kit. We noticed, the actual number of sorted cells after FACS step tends to be 20%–50% lower than the number of sorted events registered during flow cytometry. Therefore, it is recommended to validate the number and viability of FACS-sorted samples under the bright field microscope. Performing a cell count, however, reduces the number of cells available for scRNA-seq.

51. To evaluate cell viability and concentration (cell number per μL) in the FACS-sorted sample, mix 5 μL of cell suspension with 5 μL of 0.4% (w/v) Trypan Blue Solution and inspect the cells using hemocytometer under bright field microscope, or alternatively using an automated cell counter (e.g., Countess II). Cells that are dead will appear dark blue.

***Note:*** When using automated cell counter, it must be ensured that the right focus is set to count all viable cells that vary in size and shape. It is a good practice to perform a quick visual check of the automated cell counter results by inspecting the trypan blue stained cells under a bright field microscope.

52. If cell viability is below 60%, refer to troubleshooting table.
53. If cell count is below 500 cell/μL refer to troubleshooting table.
54. If cell viability is ≥80% and cell count is ≥500 cell/μL, proceed to next step.
55. Adjust the cell loading concentration to reach a desirable target of barcoded-cells (refer to Methods S1, page 24).
56. Following the manual for Chromium Controller (Methods S1, pages 25–32) to load the cells onto Chromium Chip B.
57. Perform barcoding RT reaction and amplify barcoded-cDNA by 12-cycles of PCR (refer to pages 26–34 in Methods S1 for exact reaction conditions).
58. Determine the profile of the barcoded cDNA library by diluting 1 μL sample with 5 μL water and then loading 1 μL of on an Agilent Bioanalyzer High Sensitivity chip. The expected DNA traces are shown in Figure 4.

**Figure 4:**
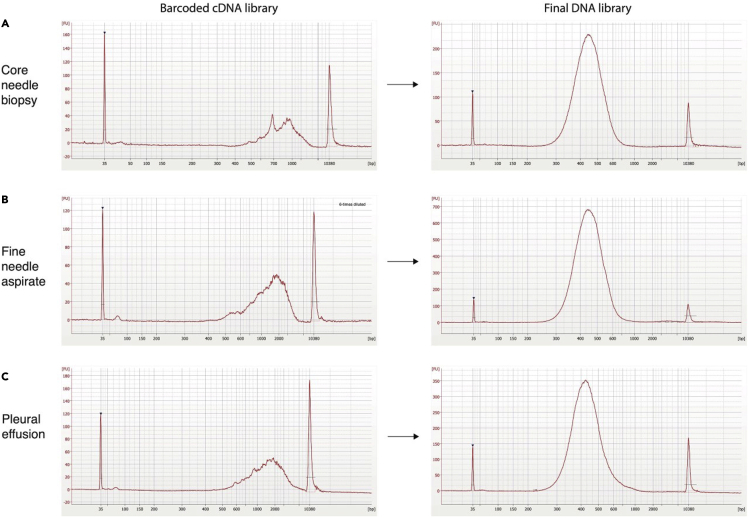
The scRNA-seq libraries prepared from the core needle biopsy, fine needle aspirate and pleural effusion samples (A) The scRNA-seq library traces of core needle biopsy. (B) The scRNA-seq library traces of fine need aspirate. (C) The scRNA-seq library traces of pleural effusion sample.
59. Following the same manual (Methods S1, pages 35–43) fragment the PCR-amplified cDNA library, ligate sequencing adaptor and perform indexing PCR.
60. After double size purification (0.6–0.8×) with SPRI beads determine the DNA concentration of the final library using Qubit 4.0 Flourometer and the dsDNA HS Assay Kit.
61. The expected DNA amount should be at ≥5.0 ng/μL. Refer to troubleshooting table if DNA amount is lower.
62. Determine the average fragment size of the final DNA library by diluting 1 μL sample with 9 μL water and then loading 1 μL of diluted DNA library on an Agilent Bioanalyzer High Sensitivity chip. The expected DNA traces are shown in Figure 4. Select the region encompassing 35–10,000 bp and record the DNA concentration.

**Pause point:** At this step libraries can be frozen at −20°C and kept indefinitely.

### Part G. Next-generation DNA sequencing

**Timing: >24 h**

Once quantified and normalized, the DNA libraries should be denatured and diluted as recommended for Illumina sequencing platforms. Refer to Illumina documentation (Denature and Dilute Libraries Guide, Document # 1000000106351 v03, Standard Loading) for denaturing and diluting libraries. Prior denaturation the DNA libraries obtained from different clinical samples (and utilizing different sample indexes) can be pooled for sequencing, yet the differences in cell number of each library should be considered.

**CRITICAL:** The DNA amount loaded onto the flow cell will greatly influence the number of clusters generated.

63. Using 10 mM Tris-HCl [pH 8.5] dilute each DNA library down to 2 nM concentration.
64. Pool individual DNA libraries into one tube.
65. Follow Illumina’s guidelines for denaturating and diluting libraries (Standard Loading).
66. When using NovaSeq 6000 S2 Reagent Kit v1.5 (100 cycles) sequence the DNA library using following cycle numbers:

   Read 1–26 cycles.

   Read i7 – 8 cycles.

   Read 2–70 cycles or longer.

67. Aim to achieve sequencing depth of 30,000–50,000 reads per cell, or 120–200 million reads per one DNA library (e.g., a pool of 8 libraries may require up to 1,600 million reads).

***Note:*** In a typical scenario, we aim to sequence ∼3,000 cells per sample at a depth such that each sample recovers ∼5,000 molecules (transcripts) per cell.

### Part H. Data pre-processing

Computational steps can be divided into data pre-processing (which ensures initial quality control) and downstream analysis. The data pre-processing includes mapping to the reference genome, filtering low-quality cells (including empty droplets and doublets) and lowly expressed genes.

**Figure 5:**
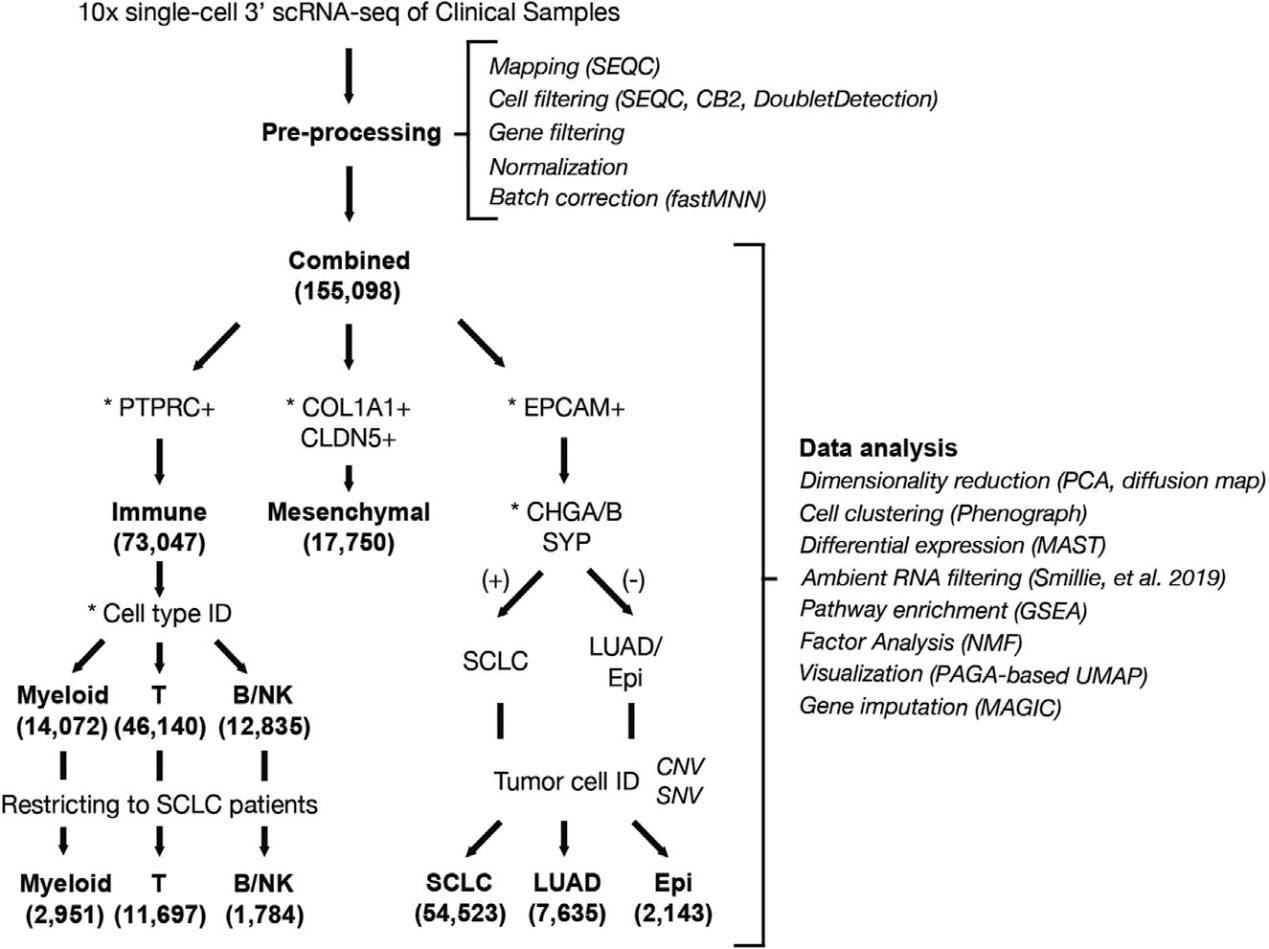
Flowchart detailing the preprocessing and analytical steps taken at iterative subsets of the single-cell cohort

The pre-processing steps of scRNA-seq are illustrated in Figure 5.

68. Mapping:
  a. Recommended option: Align fastq files from each sample to the SEQC (v0.2.11) pipeline (Azizi et al., 2018) based on the hg38 human genome reference and Ensembl 85 gene annotations using default parameters for the 10× single-cell 3′ library. The SEQC pipeline performs read alignment, multi-mapping read resolution, as well as cell barcode and UMI correction to generate a count matrix (cells × genes).
    The SEQC pipeline is available for download here. A dockerized version is also available here.
  b. Alternative option: Align fastq files from each sample to the Cell Ranger pipeline based on the hg38 human genome reference and default parameters for the 10× single-cell 3′ library.
69. **Cell filtering**: Initial cell filtering using SEQC based on the following criteria:
  a. True cells are distinguished from empty droplets based on the cumulative distribution of total molecule counts.
  b. Cells with a high fraction of mitochondrial molecules are filtered (> 20%).
  c. Cells with low library complexity are filtered (cells that express very few unique genes). This step is performed by regressing the number of genes detected per cell against the number of molecules in the same cell. Cells are excluded if the corresponding residual is greater than 3 standard deviations below the mean (Azizi et al., 2017).
70. **Empty droplet filtering** using CB2 (Ni et al., 2020)**.** Parameter “lower” set at 100 to estimate the background distribution of ambient RNA and an FDR threshold of 0.01 for calling real cells (Ni et al., 2020).
71. **Doublets filtering** using DoubletDetection package (https://doi.org/10.5281/zenodo.2658729) and default parameters? Alternatively, one may use Scrublet (Wolock et al., 2019).
72. **Gene filtering**: Retain genes expressed in >10 cells for further analysis.
73. **Normalization/transformation**: Normalize count matrix by library size, scale by median library size, and log2-transform with a pseudocount of 0.1.
74. **Batch correction**: Perform batch correction in the combined dataset of clinical samples using fastMNN (Haghverdi et al., 2018) with cosine distance applied to the log2 transform of the library-size normalized count matrix with pseudocount of 1, reduced to the top 50 PCs. We favor fastMNN due to the ability to perform hierarchical merging among samples. In particular, we first merge samples from the same patient, then from the same histology, with samples containing a greater number of cells merged first.
75. **Batch correction evaluation**: Evaluate the effect of batch correction using an entropy-based measure that quantifies how much normalized expression mixes across patients (Azizi et al., 2018). For that purpose, construct a k-nearest neighbors’ graph (k=30) from the normalized dataset using Euclidean distance and compute the fraction of cells *q*_*T*_ derived from each tumor sample *T* in the neighborhood of each cell *j.* Calculate the Shannon entropy *H*_*j*_ of sample frequencies within each cell’s neighborhood as:

$$H_{j}=\;\underset{T}{\sum }-q_{T}\;log\;q_{T}$$

Higher entropy indicates that the most similar cells come from a well-mixed set of tumors, whereas lower entropy indicates that most similar cells derive from the same tumor.

**CRITICAL:** We do not recommend performing downstream batch correction in subset compartments of coarse cell types out of concern of over-correcting tumor phenotypes.

### Part I. Data analysis

Following initial data pre-processing, downstream analysis focuses on dimensionality reduction, clustering and trajectory analysis, cell typing, factor analysis, differential expression, and gene pathway analysis, among others. These steps are illustrated in Figure 5. Computational scripts and notebooks are available here.

76. Dimensionality reduction:
  Principal component analysis (PCA):
  a. Perform PCA on the normalized expression matrix, implemented in Scanpy (Wolf et al., 2018).
  b. Retain the top PCs based on the kneepoint of variance explained.
    Diffusion map:
  c. Construct a diffusion map based on the top retained PCs, implemented in scanpy (Wolf et al., 2018).
  d. Retained the top diffusion components (DCs) prior to the largest eigengap, defined as the difference between two consecutive eigenvalues.
77. Cell clustering: These clusters represent discrete cell types or cell states in the single-cell data.
  a. Cluster cells using Phenograph algorithm (Levine et al., 2015) based on the top retained PCs over a range of values for the parameter *k* (number of neighbors in the knn-graph) to ensure that subsequent cell typing is consistent.
  b. Choose *k* from the window where the adjusted Rand index between clusters is consistently highest. In general, we found that *k* = 30 provides stable clusters in all cell compartments, with the exception of the T-cell compartment where we used *k* = 40.
78. **Differential expression:** Down-sample cells to homogenize cell sampling per batch prior to differential expression.
  a. Partition cells from each cluster into 10 equally-sized bins based on cell complexity (number of genes expressed) for subsequent downsampling.
    ***Note:*** This partitioning to downsample within each bin will be different for different datasets.
  b. Subsample from each bin to at most *k* cells per sample, where *k* is the median sample size, such that cell complexity distribution is consistent across samples.
  c. Use MAST (version 1.8.2) (Finak et al., 2015) to perform differential expression between 1) each SCLC subtype vs rest, 2) SCLC-A vs SCLC-N cells, and 3) each unsupervised cluster vs rest.
  d. Construct the regression model based on cellular detection rate (cngeneson, or number of genes detected per sample), tissue status (primary vs LN vs distant metastasis), and treatment status (naive vs most recently chemo-treated vs most recently immunotherapy-treated), as follows:
    $$Y_{i,j}\text{ ∼ }condition\;+\;tissue\;+\;treatment\;+\;cngeneson$$
    where condition represents the condition of interest and *Y*_*i*_ is the expression level of gene *i* in cells in cluster *j*, transformed by natural logarithm with a pseudocount of 1.
  e. Filter differentially expressed genes (DEGs) for significance based on Bonferroni-adjusted p-value < 0.05 and absolute log fold-change > 0.3.
79. **Ambient RNA filtering**: Identify and remove candidate DEGs that could represent ambient RNA following a stepwise, regression-based approach (Smillie et al., 2019):
  a. Fit an initial Loess regression on gene expression for each general cell type (ingroup) vs all other cells (outgroup).
  b. Bin genes by expression (number of bins = 25) and consider the 50 genes with the most negative residuals per bin.
  c. Fit a second linear regression to genes with the most negative residuals.
  d. Consider genes with residuals for the second regression that are < 2 as ambient RNA.
80. Gene pathway enrichment:
  a. Calculate gene ranks using -log(p-value)∗log fold change based on MAST (Finak et al., 2015) differential expression, described above.
  b. Input gene ranks into pre-ranked GSEA, as implemented by the R package fGSEA (Korotkevich et al., 2016) using 10,000 permutations, based on a curated set of pathways from MSigDB v 7.1 (Subramanian et al., 2005), as provided in Chan et al. *Cancer Cell*, 2021.
  c. Consider pathways with Benjamini-Hochberg adjusted p-values < 0.1 to be significant, following typical significance thresholds used in GSEA.
81. **Factor analysis**: This approach excels in settings of continuous phenotypes that are less amenable to cell clustering. Here, cells and genes are projected into the same lower-dimensional space. The resulting latent factors are associated with weights or loadings for each cell and each gene. These cell and gene loadings can be used to associate gene programs to different cells.
  a. Use non-negative matrix factorization (NMF) implemented in scikit-learn with tolerance for stopping condition 10^−4^ and maximum number of iterations 500
  b. Select the number of factors *k*.
    i. Perform NMF over a range of *k* from 5 to 100
    ii. Calculate the reconstruction error for each NMF for a given *k.* Reconstruction error is the Frobenius norm of the matrix difference between the observed gene expression matrix and the reconstructed matrix.
    iii. Select a final value of *k* based on the kneepoint of the log2 reconstruction error.
    iv. Ensure robustness to value of *k* based on Pearson’s correlation between cell loadings (where robust factorization approaches correlation of 1 for different values of *k*).
    v. We found *k* = 30 to be robust.
  c. Annotate factors.
    i. Scale gene loadings by standard deviation across genes.
    ii. Z-scored across factors.
    iii. Annotate each factor by genes with the highest loadings. The top genes for each factor can be identified using the ExtractTopFeatures function in the CountClust package (Dey et al., 2017), which extracts the top features that maximally separate each factor from others using a Kullback-Leibler divergence-based method.
82. Single-cell visualization:
  a. Perform partition-based graph abstraction (PAGA) implemented in the scanpy package using Phenograph clusters. This step provides initialization for subsequent UMAP step.
  b. Plot UMAP projections (McInnes et al., 2018) as lower dimensional representations using knn = 15, min_dist = 0.3–0.5, and init_pos = ‘paga’.
  c. To visualize gene expression, we perform gene imputation using MAGIC (knn = 30, t=3) (Dijk et al., 2018).
83. **Cell typing:** A hierarchical strategy for cell type identification proceeds first at coarse resolution (epithelial versus immune) and then fine resolution (basal versus NE cell).
  a. **Coarse cell typing**: At the coarse level, cell typing based solely on predominant expression of key gene markers below should suffice.
    i. Immune cells (*PTPRC*).
    ii. Epithelial cells (*EPCAM*).
    iii. Fibroblasts (*COL1A1*).
    iv. Endothelial (*CLDN5*).
  b. **Fine cell typing**: At the fine level, cell typing is improved using cell type-specific gene sets rather than any single gene marker. Refer to Table S1 for list of marker gene sets per cell type.
    i. Subset data and repeat steps 68–82 within each coarse cell-type compartment (without batch correction).
      ***Note:*** The analysis in the immune cell compartment benefits from Z-scoring the expression matrix prior to constructing the PCA. While the Batch Correction step can be repeated, we generally prefer not to batch correct if possible out of concern for overcorrecting biological signal and removing true tumor heterogeneity.
    ii. Z-score the expression matrix.
    iii. Use the score_genes function in scanpy, which calculates the average expression of each cell type-specific gene set and subtracts from the average expression of a reference set of genes.
    iv. Z-score the subsequent cell-type scores for comparison and choose the maximum cell-type score.
    v. Smooth cell type labels by choosing the most frequent cell type label per cluster.
  c. **Consistency check:** Assess consistency of cell typing with key cell-type markers individually:
    i. Within *EPCAM*+ epithelial cells.
          Neuroendocrine (CHGA, CHGB, NCAM1, SYP, ASCL1, ASCL2, BEX1).
          Non-neuroendocrine.
            Alveolar epithelial cells I (AGER, CLIC5, PDPN).
            Alveolar epithelial cells II (SFTPC, SFTPD, MUC1, GATA6).
            Basal cells (KRT5, TP63, AQP3, DAPL1).
            Ciliated cells (FOXJ1, CCDC78, TUBB1).
            Club cells (SCGB1A1, SCGB3A2, CCKAR).
            Hepatocytes (TF, CYP3A4, HP, ALB).
            Ionocytes (FOXI1, ASCL3, CTFR).
            Mucinous cells (MUC5AC, MUC5B, SPDEF).
            Tuft cells (POU2F3, ASCL2, DCLK1).
    ii. Within *PTPRC*+ immune cells.
          B-cells (CD19, MS4A1, CD79A).
          Plasma cells (CD79A, SDC1, MZB1, CD27).
          T-cells (CD2, CD3D, CD3E).
          Natural killer cell (NCAM1, NCR1, NKG7, KLRC1, GNLY).
          Macrophage/monocytes (*CD14, FCGR3A, ITGAM, ITGAX*).
          Neutrophils (*CSF3R, NAMPT, FCGR3B*).
          cDC (*CD1C, PPA1, LSP1, CSF2RA*).
          pDC (*GZMB, JCHAIN, IRF7, LILRA4*).
      ***Note:*** DEGs and enriched pathways per cluster as well as factor analysis can help check consistency.

84. Cancer cell identification.
  a. Cluster cells in the epithelial compartment (following details in step 77).
    i. Resulting clusters represent possible cancer cell clusters.
    ii. One can also include mesenchymal cells if there is a strong suspicion of *EPCAM-* cancer cells that have undergone epithelial-mesenchymal transformation (EMT).
    iii. Exclude any clusters that contain cells derived from normal adjacent lung samples.
  b. Leverage single nucleotide variants (SNVs).
    i. Clinical DNA sequencing of a targeted panel of 468 genes was previously performed on matched bulk tumors using the MSK IMPACT platform (Cheng et al., 2015). Integration of the matched point mutational profile of the bulk tumor can facilitate cancer cell detection.
    ii. Obtain the variant calls (base changes and genomic coordinates) from matched bulk DNA-sequencing.
    iii. Identify UMIs from the aligned scRNA-seq bam file of each sample (with reads collapsed to UMI) that match the same variant calls from bulk DNA-sequencing.
      ***Note:****de novo* SNV detection at single-cell level could be used instead but is outside the scope of this STAR protocol.
    iv. Tally the proportion of UMIs that call SNVs in each epithelial cluster.
    v. Tally the proportion of UMIs that call SNVs in normal immune and mesenchymal cells. This measure provides a negative control that represents the rate of detection from ambient RNA.
    vi. Calculate the Fisher’s p-value as a measure of enrichment for UMIs that call SNVs.
    vii. Adjust the Fisher’s p-value by Bonferroni calculation for multiplicity with a significance threshold of < 0.05.
  c. Leverage copy number variants (CNVs).
    i. Use InferCNV (Patel et al., 2014) with the following parameters:
          Sliding window of 200 genes.
          Diploid mean and standard deviation based on available normal adjacent tumor samples.
          Consider at least two standard deviations from diploid mean to represent a CNV.
    ii. Calculate CNV burden using two alternative measures.
          Fraction of the genome affected by CNV.
          Pearson’s correlation between single-cell and bulk CNV profiles.
  d. Compare CNV burden between tumor and normal samples.
    i. Typically, tumor samples will display a bimodal distribution, with a lower peak corresponding to normal stromal cells and a higher peak corresponding to mutated cancer cells. Normal samples will display a unimodal distribution.
    ii. Based on our normal samples in the SCLC atlas, we identified cancer cells using a threshold of >10% fraction of genome altered and Pearson’s correlation to bulk CNV profile rho >0.2.
85. **Small cell lung cancer (SCLC) subtyping**: We recommend subtype classification based on phenotype rather than only the canonical set of four transcription factors (i.e., *ASCL1, NEUROD1, POU2F3,* and *YAP1*).
  a. Feature selection: We recommend restricting analysis to a set of genes that excludes genes that are not subtype-specific. For instance, in our initial analysis of the SCLC atlas, we restricted to DEGs between each SCLC-subtype (SCLC-A, SCLC-N, SCLC-P, SCLC-Y) vs rest previously identified from bulk RNA-seq. We also exclude genes from cell cycle, hypoxia, and apoptosis pathways that are non-specific to SCLC subtype and might confound classification. These filtered genes included pathways from REACTOME_CELL_CYCLE_MITOTIC, REACTOME_MITOTIC_G1_G1_S_PHASES, HALLMARK_G2M_CHECKPOINT, HALLMARK_HYPOXIA, HALLMARK_APOPTOSIS downloaded from MSigDB. As we refined DEGs between subtypes in our single-cell atlas, one could now restrict analysis to these DEGs for subtype classification in any new dataset.
  b. Identify the most representative cells for each SCLC subtype.
    i. Identify the top 30 overexpressed DEGs per SCLC subtype from the bulk RNA-seq reference.
    ii. Calculate the average Z-score over this gene set for each cell.
    iii. Label the top 100 highest scoring cells for each SCLC subtype as training examples for subsequent subtype classification.
  c. Construct a diffusion map retaining the top DCs based on eigengap, as above.
  d. Use Phenograph classifier to perform Markov absorption classification in a Jaccard similarity graph constructed from the top DCs. This step will result in per-cell probabilities for each SCLC subtype, which can be considered a deconvolution of mixed subtypes.
  e. Assign the most likely SCLC subtype by the maximum probability.

## Expected outcomes

**Figure 6:**
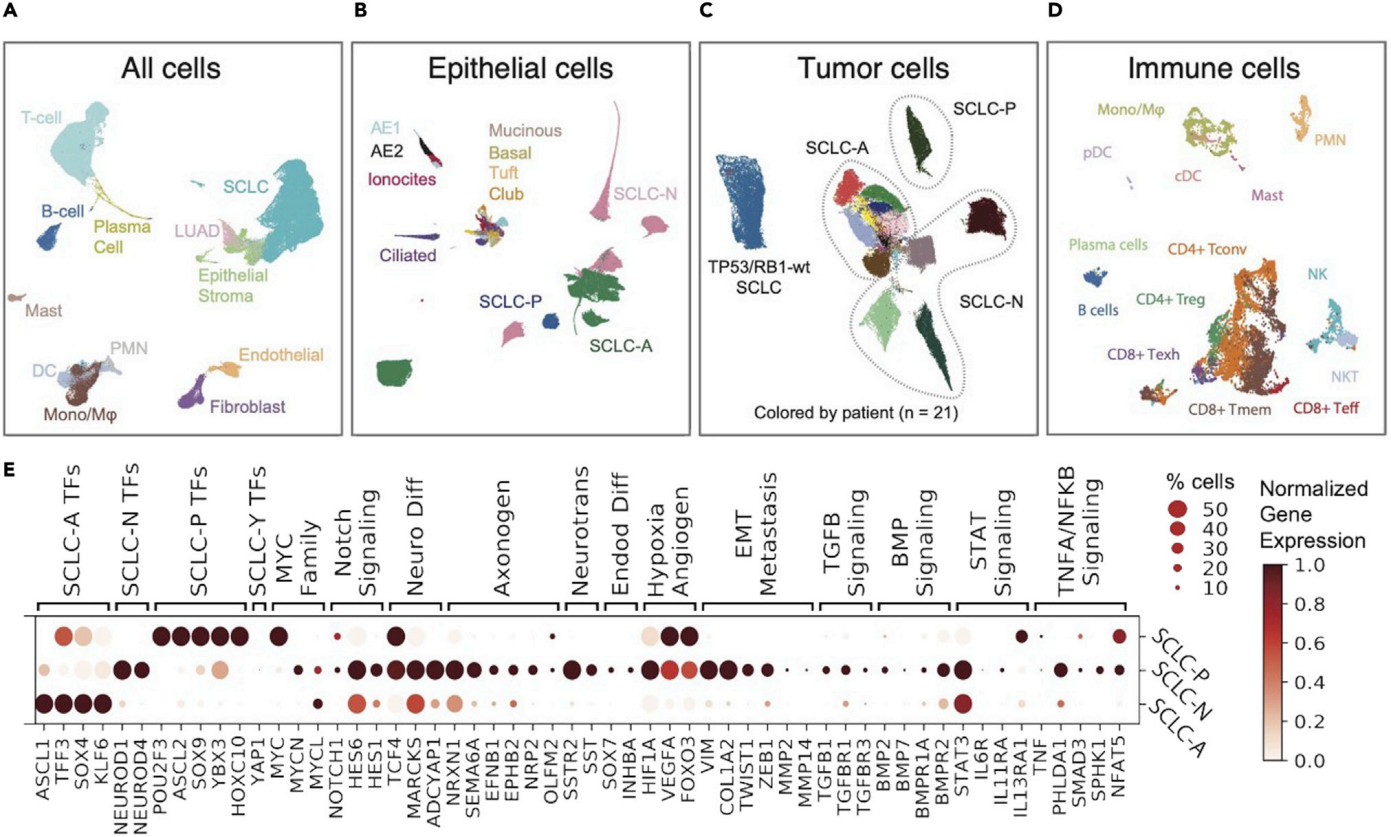
The single-cell transcriptional atlas of human lung tumor (A) UMAP projection of the human lung cohort at the global level (n = 155,098 cells). (B) UMAP projection of the epithelial cells at the global level (n = 64,301 cells). (C) UMAP projection of the small cell lung cancer (n = 54,523 cells). (D) UMAP projection of the immune cells from small cell lung cancer samples (n = 16,475 cells). (E) Dot plot show selected differentially expressed genes between each SCLC subtype versus the rest, mean normalized expression and percent of cells expressing a given gene. SCLC - small cell lung cancer; LUAD – lung adenocarcinoma; T_conv_ - conventional T-cell; T_reg_ - regulatory T-cell; T_eff_ - effector T-cell; T_mem_ - memory T-cell; Mono/Mφ = monocyte/macrophage; PMN = neutrophil; cDC = conventional dendritic cell; pDC = plasmacytoid dendritic cell.

Following the protocol reported here approximately 100,000 to a few millions of cells can be obtained from resection samples (>100 mg of tissue), or pleural fluids (≥250 mL volume). From core needle biopsies or fine needle aspirates (10–50 mg of tissue) one could expect to obtain 15,000–60,000 of FACS-sorted cells. The cell viability, after FACS, will vary between 71 and 98% (median 80%), when evaluated by trypan blue dye exclusion assay. Sequencing samples at depth 120–200 M reads (∼40,000 reads per cell), one could expect to recover ∼5,000 unique molecules (transcripts) per cell, and detect 2100 expressed genes, on average. The unique mapping is expected to be close to 80%. The scRNA-seq data analysis procedure described here provides a guideline for constructing human tumor cell atlases, similar to the one presented in Figure 6.

Arguably, the high intrinsic heterogeneity of tumor samples makes it difficult to predict the number of cells to be obtained from a given clinical sample. Different tumor types tend to display different cellular compositions (i.e., lung adenocarcinomas usually contain 5%–20% of cancer cells while in small cell lung carcinomas the cancer cell fraction may be up to 50%–90% of total cells). Nonetheless, the protocol detailed here was successfully applied to profile the single transcriptomes of fresh small cell lung cancer (SCLC), lung adenocarcinoma (LUAD) and tumor-adjacent normal lung samples, acquired from untreated and treated patients (Chan et al., 2021). In addition, the clinical samples processed included primary tumors, regional lymph node metastases, and distant metastases (liver, adrenal gland, axilla, and pleural effusion), suggesting a broad utility of the reported protocol.

## Limitations

As with most protocols, dissociation of human tissue specimens based on mechanical and enzymatic treatment has the potential to introduce substantial cell subpopulation biases if a given intra-tumoral cell subset is particularly sensitive to temperature, enzymatical or mechanical stress. The epithelial cells are more sensitive to dissociation and are more fragile than the suspension (immune) cells. Therefore, this protocol is only applicable with certain guarantee of success to freshly collected samples. Processing of samples that have been processed over 2 h of post-acquisition, or have been transported under suboptimal conditions, will likely result in significantly reduced cell viability, compromised transcriptome integrity, and undesirable biological or technical artifacts. The rule of thumb: the quicker the isolation, the better the status of the cells.

Samples collected while the patient is on-treatment will show highly variable cell viability and recovery. In addition, when working with small size biospecimens such as core needle or fine needle aspirate, the cancer cell recovery can be low (n ≤ 10,000) and below the recommendations for scRNA-seq using 10× Chromium controller. In such scenarios, the FACS-based enrichment of CD45-negative and CD45-positive cells into two separate fractions provides a flexible option to later mix tumor and immune cells at a desirable ratio in order to obtain an adequate concentration of cells (n ≥ 20,000) for scRNA-seq.

Other variables can also influence the results. These include biological variables such a tissue origin, as RNAses are differentially expressed in distinct tumor types; or clinical variables, such as specimen collection performance, patient drug treatment history, tissue necrosis, ischemic time, etc. These experimental variables can significantly affect the cell viability in the biospecimen. Evidently, the starting amount of tissue or fluid is also an important variable and will most likely correlate with the number of cells finally obtained.

## Troubleshooting

### Problem 1

Poor tissue dissociation (steps #6 or #17).

### Potential solution

Lung tissues that are rich in extracellular matrix may comprise limited number of cells available for isolation. Some biospecimens, may contain fats that tend to reduces the efficiency of enzymatic digestion and tissue dissociation. One straightforward option is extending digestion time, however, at the risk of reducing cell viability.

### Problem 2

Insufficient number of cells obtained for FACS (step #45).

### Potential solution

There are multiple reasons why cell recovery might be insufficient. The quality of biospecimens are difficult to predict and unfortunately some samples that do not pass QC will need to be discarded. Avoid necrotic tissue samples or biospecimens that took >2 h to transfer to the laboratory. If possible, increase the size of biospecimen. Ensure that during centrifugation steps the cell pellet remains undisturbed and is not removed accidentally along with the supernatant. In some cases, the cell pellets might very small and loosely packed, and therefore extra care must be taken when aspirating the supernatant.

### Problem 3

**Figure 7:**
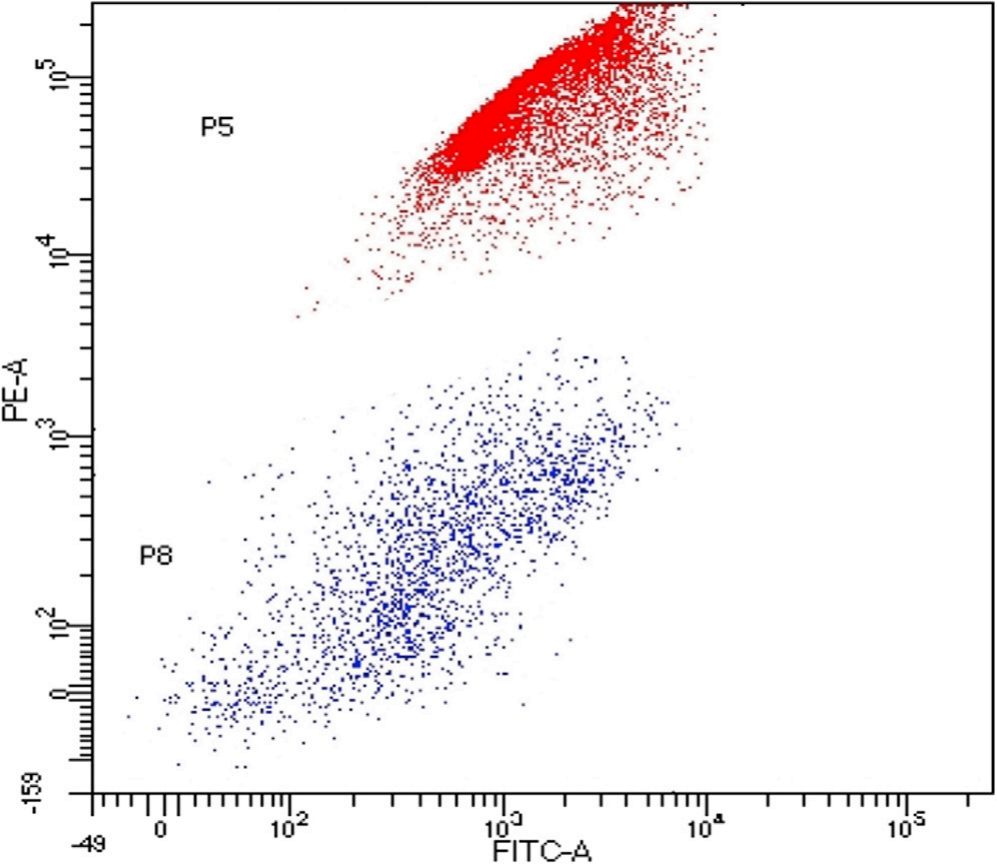
Illustrative FACS plot with inefficient separation of dead and live cells Y-axis indicates the fluorescence intensity of anti-CD45 staining (PE-A) and X-axis represents the fluorescence intensity of calcein dye (FITC-A).

Inefficient separation of live and dead cells by Calcein-AM staining in the flow cytometry plot (Figure 7), while performing FACS (step #45).

### Potential solution

Calcein-AM is a cell-permeant fluorogenic substrate that is hydrolyzed by intracellular esterases to a green-fluorescent product, calcein. The yield of calcein and thus the fluorescent signal intensity of stained-cells is dependent on the esterase activity. Therefore, cells that express reduced levels of esterases may require a prolonged incubation with calcein-AM to ensure that sufficient amount of calcein has accumulated within cell cytoplasm. Another potential solution is to perform calcein-AM staining and tissue dissociation simultaneously. In such scenario, calcein-AM reagent should be added to the GentleMACS C tube, as the higher temperature during enzymatic dissociation (37 ᵒC as compared to 4 ᵒC during staining with Ab) will increase catalytic esterase activity, leading to a higher yield of fluorescent calcein. If calcein-AM staining cannot be optimized due to limited biospecimen availability, or inefficient calcein staining is encountered while sorting an important sample, then restrictive DAPI-negative gating may facilitate the recovery of cells with acceptable viability.

### Problem 4

**Figure 8:**
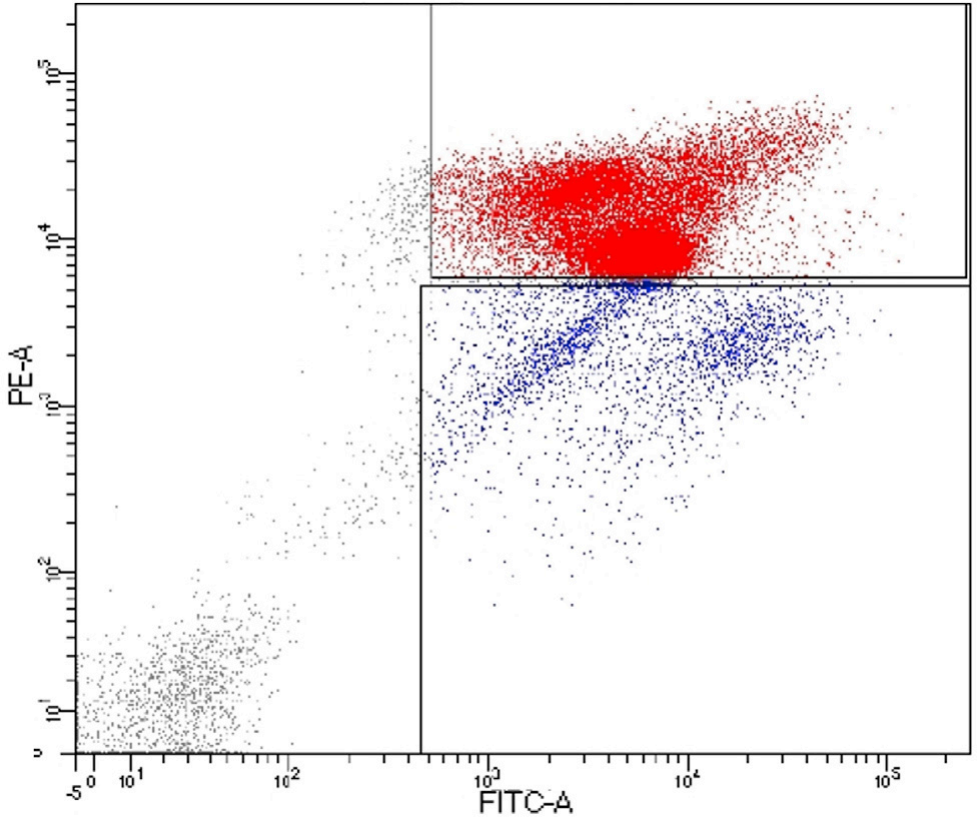
Illustrative FACS plot with inefficient separation of CD45-negative and CD45-positive cells Y-axis indicates the fluorescence intensity of anti-CD45 staining (PE-A) and X-axis represents the fluorescence intensity of calcein (FITC-A).

Inefficient separation of immune and non-immune cells in the flow cytometry plot (Figure 8), while performing FACS (step #45).

### Potential solution

In rare cases this problem may arise due to insufficient amount of CD45 antibody in the staining cocktail. Some specimens, like pleural effusions, may contain extremely high numbers of immune cells, which will sequester most of CD45 antibody available. Therefore, when processing a sample comprising a high number of cells it may be advantageous to increase CD45 antibody amount in the staining cocktail.

### Problem 5

Clogging of FACS instrument during sorting procedure (step #46).

### Potential solution

Because EDTA is absent in the sample dissociation buffer (this is to prevent potential inhibition of the reverse transcription reaction) the chance of cell clumping and clogging, increases. It may be beneficial to include filtering of the pre-sorting cell suspension through 70 μm cell strainer (more than once, if necessary) or diluting the cell suspension before FACS.

### Problem 6

The viability of post-FACS cells is below 60% (step #52).

### Potential solution

Increase the stringency of sorting gates by selecting cells that show high signal in calcein (green channel). Slow down the sorting speed (below 5,000 events/s); this will result in lower share forces acting upon cells. If possible, use FACS sheath fluid supplemented with 2.5% FBS. Keep cells at 4°C and avoid prolonged exposure to 20°C–25°C.

### Problem 7

The cell count of post-FACS sample is below 500 cells/μL (step #53).

### Potential solution

When the cell concentration is low, further processing for barcoding reverse transcription reaction depends on the total volume of the sample. If the total volume of the sample is ≥40 μL, concentrate the cells by centrifugation. Perform centrifugation at 500 g in a swinging bucket centrifuge at 4°C for 5 min, carefully remove the excess volume of supernatant until a desirable volume is left (e.g., 20 μL), gently resuspend the cell pellet and load a required number of cells on a Chromium Chip. If the total volume of the sample is ≤40 μL, one may proceed to “Part F. Droplet-based single-cell RNA-Seq” section with expectation to recover fewer cells. In latter scenario, amplify the barcoded-cDNA library by 14-cycles of PCR.

### Problem 8

The DNA amount in the final library is below 5.0 ng/μL (step #61).

### Potential solution

Perform 2–4 additional cycles of PCR on the final library and purify the resulting material with 0.6–0.8× SPRIselect Reagent Kit.

### Problem 9

Poor recovery of transcripts and genes after the sequencing (step #67).

### Potential solution

Most common cause of low transcript recovery is poor cell quality and mRNA degradation. Make sure that reagents and consumables used for scRNA-seq are free of nucleases. To extend cell viability, process samples quickly and ensure that cells are kept on ice throughout the protocol with minimal exposure to ambient temperatures. The transcript capture might be also affected by suboptimal RT reaction conditions, for instance, if tissue dissociation is performed in the presence of DNAse I, or other inhibiting factors.

### Problem 10

Undesirable batch effects (steps #73 and 74).

### Potential solution

When working with clinical samples batch effects are not uncommon. Perform **Batch correction** and **Batch correction evaluation** as explained in Part I. Data analysis section. Processing the samples using exactly the same procedure, including the time that it takes to process the biospecimens until scRNA-seq step, should minimize the technical batch effects. It might be beneficial to validate the technical batch effects of the protocol by dividing the biospecimen in two halves and then processing both in parallel following the protocol provided here. We conducted such control experiment and observed no technical batch effects.

## Resource availability

### Lead contact

Further information and requests for resources and reagents should be directed to and will be fulfilled by the lead contact, Linas Mazutis (linas.mazutis@bti.vu.lt).

### Materials availability

Not applicable.

## Data Availability

Software and tools used for the SCLC atlas and described in this STAR Protocol are open source at https://github.com/dpeerlab/SCLC_atlas-HTAN (https://doi.org/10.5281/zenodo.7035166). In collaboration with the NIH-funded HTAN Data Coordinating Center (U24), raw and processed single-cell RNA-seq data are downloadable and also available as an interactive, online platform for independent visualization and analysis at https://data.humantumoratlas.org and https://cellxgene.cziscience.com/collections/62e8f058-9c37-48bc-9200-e767f318a8ec. The accession number for the data reported in this paper is HTAN Data Portal: HTAN MSK.

## Supplemental information

Methods S1. Manual for using ChromiumTM instrument and reagent kit, related to steps 55–59

Table S1. List of marker gene sets per cell type, related to step 83
