# Supplementary material for: Protocol to dissociate, process, and analyze the human lung tissue using single-cell RNA-seq
Source: STAR Protoc. 2022 Oct 21;3(4):101776. doi: 10.1016/j.xpro.2022.101776 (PMC9597186; doi:10.1016/j.xpro.2022.101776)
Supplement: Methods S1. Manual for using ChromiumTM instrument and reagent kit, related to steps 55–59 [file mmc1.zip › Chromium manual.pdf]

CG000183 Rev B

USER GUIDE

Chromium Single Cell 3' Reagent Kits v3

FOR USE WITH

Chromium Single Cell 3' GEM, Library & Gel Bead Kit v3, 16 rxns PN-1000075
Chromium Single Cell 3' GEM, Library & Gel Bead Kit v3, 4 rxns PN-1000092
Chromium Single Cell B Chip Kit, 48 rxns PN-1000073
Chromium Single Cell B Chip Kit, 16 rxns PN-1000074
Chromium i7 Multiplex Kit, 96 rxns PN-120262

Available for use only with the indicated Chromium Controller (PN-120223;120246) or Chromium Single Cell Controller (PN-120263;120212).

10xGenomics.com

Notices

Document Number

CG000183 | Rev B

Legal Notices

© 2019 10X Genomics, Inc (10x Genomics). All rights reserved. Duplication and/or reproduction of all or any portion of this document without the express written consent of 10x Genomics, is strictly forbidden. Nothing contained herein shall constitute any warranty, express or implied, as to the performance of any products described herein. Any and all warranties applicable to any products are set forth in the applicable terms and conditions of sale accompanying the purchase of such product. 10x Genomics provides no warranty and hereby disclaims any and all warranties as to the use of any third-party products or protocols described herein. The use of products described herein is subject to certain restrictions as set forth in the applicable terms and conditions of sale accompanying the purchase of such product. A non-exhaustive list of 10x Genomics' marks, many of which are registered in the United States and other countries can be viewed at: www.10xgenomics.com/trademarks. 10x Genomics may refer to the products or services offered by other companies by their brand name or company name solely for clarity, and does not claim any rights in those third party marks or names. 10x Genomics products may be covered by one or more of the patents as indicated at: www.10xgenomics.com/patents. The use of products described herein is subject to 10x Genomics Terms and Conditions of Sale, available at www.10xgenomics.com/legal-notices, or such other terms that have been agreed to in writing between 10x Genomics and user. All products and services described herein are intended FOR RESEARCH USE ONLY and NOT FOR USE IN DIAGNOSTIC PROCEDURES.

Instrument & Licensed Software Updates Warranties

Updates to existing Instruments and Licensed Software may be required to enable customers to use new or existing products. In the event of an Instrument failure resulting from an update, such failed Instrument will be replaced or repaired in accordance with the 10x Limited Warranty, Assurance Plan or service agreement, only if such Instrument is covered by any of the foregoing at the time of such failure. Instruments not covered under a current 10x Limited Warranty, Assurance Plan or service agreement will not be replaced or repaired.

Support

Email: support@10xgenomics.com

10x Genomics

6230 Stoneridge Mall Road

Pleasanton, CA 94588 USA

Table of Contents

Introduction 4

Chromium Single Cell 3' Reagent Kits v3 5
Chromium Accessories 8
Recommended Thermal Cyclers 8
Additional Kits, Reagents & Equipment 9
Protocol Steps & Timing 11
Stepwise Objectives 12

Tips & Best Practices 15

Step 1 21
GEM Generation & Barcoding 22

1.1 Prepare Master Mix 23
1.2 Load Chromium Chip B 25
1.3 Run the Chromium Controller 26
1.4 Transfer GEMs 26
1.5 GEM-RT Incubation 27

Step 2 28
Post GEM-RT Cleanup & cDNA Amplification 29

2.1 Post GEM-RT Cleanup – Dynabeads 30
2.2 cDNA Amplification 32
2.3 cDNA Cleanup – SPRIselect 33
2.4 cDNA QC & Quantification 34

Step 3 35
3' Gene Expression Library Construction 36

3.1 Fragmentation, End Repair & A-tailing 37
3.2 Post Fragmentation, End Repair & A-tailing Double Sided Size Selection – SPRIselect 38
3.3 Adaptor Ligation 39
3.4 Post Ligation Cleanup – SPRIselect 40
3.5 Sample Index PCR 41
3.6 Post Sample Index PCR Double Sided Size Selection – SPRIselect 42
3.7 Post Library Construction QC 43

Sequencing 44

Troubleshooting 47

5.1 GEMs 48
5.2 Chromium Controller Errors 50

Appendix 51

Post Library Construction Quantification 52
Agilent TapeStation Traces 53
Oligonucleotide Sequences 54

Introduction

Chromium Single Cell 3' Reagent Kits v3
Chromium Accessories
Recommended Thermal Cyclers
Additional Kits, Reagents & Equipment
Protocol Steps & Timing
Stepwise Objectives

Chromium Single Cell 3' Reagent Kits v3

Chromium Single Cell 3' GEM, Library & Gel Bead Kit v3, 16 rxns PN-1000075

Chromium Single Cell 3' GEM Kit v3 16 rxns PN-1000077 (store at –20°C)

Chromium
Single Cell 3'
GEM Module

	#	PN
RT Reagent	1	2000086
RT Enzyme C	1	2000085
Template Switch Oligo	1	3000228
Reducing Agent B	1	2000087
Cleanup Buffer	2	2000088
cDNA Primers	1	2000089
Amp Mix	1	2000047

10xGenomics.com 10x GENOMICS

Chromium Single Cell 3' Library Kit v3 16 rxns PN-1000078 (store at –20°C)

Chromium
Single Cell 3'
Library Module

	#	PN
Fragmentation Enzyme	1	2000090
Fragmentation Buffer	1	2000091
Ligation Buffer	1	2000092
DNA Ligase	1	220110
Adaptor Oligos	1	2000094
SI Primer	1	2000095
Amp Mix	1	2000047

10xGenomics.com 10x GENOMICS

Chromium Single Cell 3' Gel Bead Kit v3, 16 rxns PN-1000076 (store at –80°C)

Chromium
Single Cell 3'
v3 Gel Beads

	#	PN
Single Cell 3' v3 Gel Beads	2	2000059

10xGenomics.com 10x GENOMICS

Dynabeads™ MyOne™ SILANE PN-2000048 (store at 4°C)

	#	PN
Dynabeads MyOne SILANE	1	2000048

Chromium Single Cell 3' GEM, Library & Gel Bead Kit v3, 4 rxns PN-1000092

Chromium Single Cell 3' GEM Kit v3 4 rxns PN-1000094 (store at –20°C)

Chromium
Single Cell 3'
GEM Module

	#	PN
RT Reagent	1	2000086
RT Enzyme C	1	2000102
Template Switch Oligo	1	3000228
Reducing Agent B	1	2000087
Cleanup Buffer	1	2000088
cDNA Primers	1	2000089
Amp Mix	1	2000103

10xGenomics.com 10x GENOMICS

Chromium Single Cell 3' Library Kit v3 4 rxns PN-1000095 (store at –20°C)

Chromium
Single Cell 3'
Library Module

	#	PN
Fragmentation Enzyme	1	2000104
Fragmentation Buffer	1	2000091
Ligation Buffer	1	2000092
DNA Ligase	1	220131
Adaptor Oligos	1	2000094
SI Primer	1	2000095

10xGenomics.com 10x GENOMICS

Chromium Single Cell 3' Gel Bead Kit v3, 4 rxns PN-1000093 (store at –80°C)

Chromium
Single Cell 3'
v3 Gel Beads

	#	PN
Single Cell 3' v3 Gel Beads (4 rxns)	1	2000059

10xGenomics.com 10x GENOMICS

Dynabeads™ MyOne™ SILANE PN-2000048 (store at 4°C)

	#	PN
Dynabeads MyOne SILANE	1	2000048

Chromium Chip B Single Cell Kit, 48 rxns PN-1000073 (store at ambient temperature)

Chromium
Partitioning Oil

	#	PN
Partitioning Oil	6	220088

Chromium
Recovery Agent

	#	PN
Recovery Agent	6	220016

Chromium
Chip B & Gaskets

	#	PN
Chip B Single Cell	6	2000060
Gasket, 6-pack	1	370017

10xGenomics.com

Chromium Chip B Single Cell Kit, 16 rxns PN-1000074 (store at ambient temperature)

Chromium
Partitioning Oil

	#	PN
Partitioning Oil	2	220088

Chromium
Recovery Agent

	#	PN
Recovery Agent	2	220016

Chromium
Chip B & Gaskets

	#	PN
Chip B Single Cell	2	2000060
Gasket, 2-pack	1	3000072

10xGenomics.com

Chromium i7 Multiplex Kit, 96 rxns PN-120262 (store at –20°C)

Chromium
i7 Multiplex Kit

	#	PN
Chromium i7 Sample Index Plate	1	220103

Chromium Accessories

Product	Part Number (Kit)	Part Number (Item)
10x Vortex Adapter	120251	330002
10x Vortex Clip	120253	230002
10x Chip Holder	120252	330019
10x Magnetic Separator	120250	230003

Recommended Thermal Cyclers

Thermal cyclers used must support uniform heating of 100 µl emulsion volumes.

Supplier	Description	Part Number
BioRad	C1000 Touch Thermal Cycler with 96-Deep Well Reaction Module	1851197
Eppendorf	MasterCycler Pro	North America 950030010<br>International 6321 000.019
Thermo Fisher Scientific	Veriti 96-Well Thermal Cycler	4375786

Additional Kits, Reagents & Equipment

The items in the table below have been validated by 10x Genomics and are highly recommended for the Chromium Single Cell 3' protocol. Substituting materials may adversely affect system performance.

Supplier	Description		Part Number (US)
**Plastics**			
Eppendorf	PCR Tubes 0.2 ml 8-tube strips<br>DNA LoBind Tubes, 1.5 ml<br>DNA LoBind Tubes, 2.0 ml	Choose either Eppendorf, USA Scientific or Thermo Fisher Scientific PCR 8-tube strips.	951010022<br>022431021<br>022431048
USA Scientific	TempAssure PCR 8-tube strip		1402-4700
Thermo Fisher Scientific	MicroAmp 8-Tube Strip, 0.2 ml<br>MicroAmp 8 -Cap Strip, clear		N8010580<br>N8010535
Rainin	Tips LTS 200UL Filter RT-L200FLR<br>Tips LTS 1ML Filter RT-L1000FLR<br>Tips LTS 20UL Filter RT-L10FLR		30389240<br>30389213<br>30389226
**Kits & Reagents**			
Thermo Fisher Scientific	Nuclease-free Water		AM9937
	Low TE Buffer (10 mM Tris-HCl pH 8.0, 0.1 mM EDTA)		12090-015
Millipore Sigma	Ethanol, Pure (200 Proof, anhydrous)		E7023-500ML
Beckman Coulter	SPRIselect Reagent Kit		B23318
Bio-Rad	10% Tween 20		1662404
Ricca Chemical Company	Glycerin (glycerol), 50% (v/v) Aqueous Solution		3290-32
Qiagen	Qiagen Buffer EB		19086
**Equipment**			
VWR	Vortex Mixer<br>Divided Polystyrene Reservoirs		10153-838<br>41428-958
Eppendorf	Eppendorf ThermoMixer C<br>Eppendorf SmartBlock 1.5 ml, Thermoblock for 24 reaction vessel<br>(alternatively, use a temperature-controlled Heat Block)		5382000023<br>5360000038
Rainin	Pipet-Lite Multi Pipette L8-50XLS+<br>Pipet-Lite Multi Pipette L8-200XLS+<br>Pipet-Lite Multi Pipette L8-10XLS+<br>Pipet-Lite Multi Pipette L8-20XLS+<br>Pipet-Lite LTS Pipette L-2XLS+<br>Pipet-Lite LTS Pipette L-10XLS+<br>Pipet-Lite LTS Pipette L-20XLS+<br>Pipet-Lite LTS Pipette L-100XLS+<br>Pipet-Lite LTS Pipette L-200XLS+<br>Pipet-Lite LTS Pipette L-1000XLS+		17013804<br>17013805<br>17013802<br>17013803<br>17014393<br>17014388<br>17014392<br>17014384<br>17014391<br>17014382

Additional Kits, Reagents & Equipment

The items in the table below have been validated by 10x Genomics and are highly recommended for the Chromium Single Cell 3' protocol. Substituting materials may adversely affect system performance.

Supplier	Description		Part Number (US)
**Quantification & Quality Control**			
Agilent	2100 Bioanalyzer Laptop Bundle<br>High Sensitivity DNA Kit<br>4200 TapeStation<br>High Sensitivity D1000 ScreenTape/Reagents<br>High Sensitivity D5000 ScreenTape/Reagents	Choose Bioanalyzer, TapeStation, or Qubit based on availability & preference.	G2943CA<br>5067-4626<br>G2991AA<br>5067-5592/ 5067-5593<br>5067-5584/ 5067-5585
Thermo Fisher Scientific	Qubit 4.0 Flourometer<br>Qubit dsDNA HS Assay Kit		Q33226<br>Q32854
Advanced Analytical	Fragment Analyzer Automated CE System - 12 cap<br>Fragment Analyzer Automated CE System - 48/96 cap<br>High Sensitivity NGS Fragment Analysis Kit		FSv2-CE2F<br>FSv2-CE10F<br>DNF-474
KAPA Biosystems	KAPA Library Quantification Kit for Illumina Platforms		KK4824

Protocol Steps & Timing

Day	Steps	Timing	Stop & Store
	**Cell Preparation**		
2 h	Dependent on Cell Type	~1-1.5 h	
	**Step 1 – GEM Generation & Barcoding**		
	1.1 Prepare Reaction Mix	20 min	
	1.2 Load Chromium Single Cell B Chip	10 min	
	1.3 Run the Chromium Controller	8.5 min	
	1.4 Transfer GEMs	3 min	
	1.5 GEM-RT Incubation	55 min	STOP 4°C ≤72 h or –20°C ≤1 week
4 h	**Step 2 – Post GEM-RT Cleanup & cDNA Amplification**		
	2.1 Post GEM RT-Cleanup – Dynabead	45 min	
	2.2 cDNA Amplification	40 min	STOP 4°C ≤72 h or –20°C ≤1 week
	2.3 cDNA Cleanup – SPRIselect	20 min	STOP 4°C ≤72 h –20°C ≤4 weeks
	2.4 cDNA QC & Quantification	50 min	
6 h	**Step 3 – 3' Gene Expression Library Construction**		
	3.1 Fragmentation, End Repair & A-tailing	50 min	
	3.2 Post Fragmentation, End Repair & A-tailing Double Sided Size Selection – SPRIselect	30 min	
	3.3 Adaptor Ligation	25 min	
	3.4 Post Ligation Cleanup- SPRIselect	20 min	
	3.5 Sample Index PCR	40 min	STOP 4°C ≤72 h
8 h	3.6 Post Sample Index PCR Double Sided Size Selection- SPRIselect	30 min	STOP 4°C ≤72 h or -20°C long term
	3.7 Post Library Construction QC	50 min	

Stepwise Objectives

The Chromium Single Cell Gene Expression Solution upgrades short read sequencers to deliver a scalable microfluidic platform for 3' digital gene expression by profiling 500-10,000 individual cells per sample. GemCode Technology samples a pool of ~3,500,000 10x Barcodes to separately index each cell's transcriptome. It does so by partitioning thousands of cells into nanoliter-scale Gel Beads-in-emulsion (GEMs), where all generated cDNA share a common 10x Barcode. Libraries are generated and sequenced from the cDNA and 10x Barcodes are used to associate individual reads back to the individual partitions.

This document outlines the protocol for generating Single Cell 3' Gene Expression libraries from single cells.

Single Cell 3' v3 Gel Beads

In addition to the poly(dT) primer that enables the production of barcoded, full-length cDNA from poly-adenylated mRNA, the Single Cell 3' v3 Gel Beads also include two additional primer sequences (Capture Sequence 1 and Capture Sequence 2), that enable capture and priming of Feature Barcoding technology compatible targets or analytes of interest.

Only the poly(dT) primers are used in this protocol for generating Single Cell 3' Gene Expression libraries.

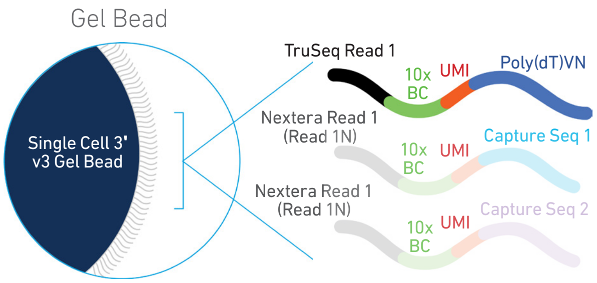

Step 1 GEM Generation & Barcoding

GEMs are generated by combining barcoded Single Cell 3' v3 Gel Beads, a Master Mix containing cells, and Partitioning Oil onto Chromium Chip B. To achieve single cell resolution, cells are delivered at a limiting dilution, such that the majority (~90-99%) of generated GEMs contain no cell, while the remainder largely contain a single cell.

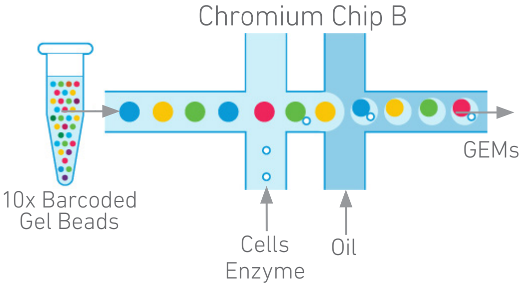

Step 1
GEM Generation & Barcoding

Immediately following GEM generation, the Gel Bead is dissolved, primers are released, and any co-partitioned cell is lysed. Primer containing:

- an Illumina TruSeq Read 1 (read 1 sequencing primer)
- 16 nt 10x Barcode
- 12 nt unique molecular identifier (UMI)
- 30 nt poly(dT) sequence

are mixed with the cell lysate and a Master Mix containing reverse transcription (RT) reagents. Incubation of the GEMs produces barcoded, full-length cDNA from poly-adenylated mRNA.

Inside individual GEMs

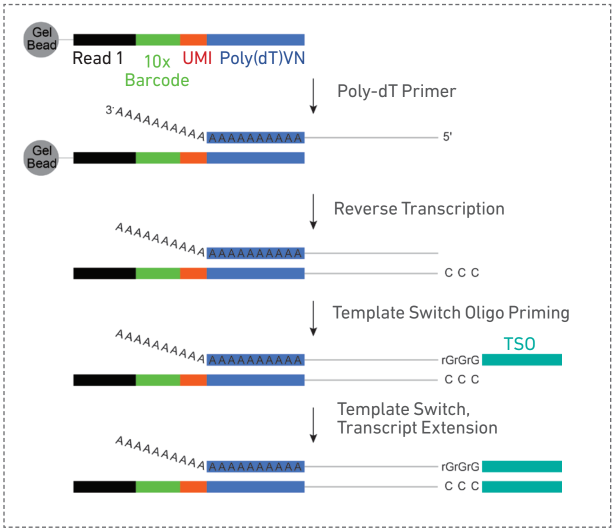

Step 2
Post GEM-RT Cleanup & cDNA Amplification

After incubation, GEMs are broken and pooled fractions are recovered. Silane magnetic beads are used to purify the first-strand cDNA from the post GEM-RT reaction mixture, which includes leftover biochemical reagents and primers. Barcoded, full-length cDNA is amplified via PCR to generate sufficient mass for library construction.

Pooled cDNA amplification

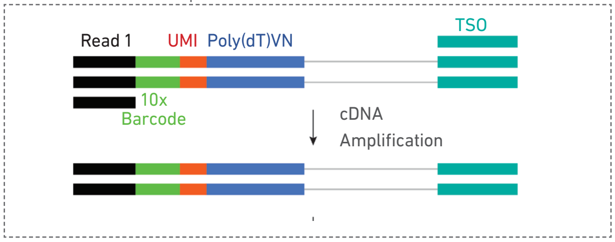

Step 3
3' Gene Expression Library Construction

Enzymatic fragmentation and size selection are used to optimize the cDNA amplicon size. TruSeq Read 1 (read 1 primer sequence) is added to the molecules during GEM incubation. P5, P7, a sample index, and TruSeq Read 2 (read 2 primer sequence) are added via End Repair, A-tailing, Adaptor Ligation, and PCR. The final libraries contain the P5 and P7 primers used in Illumina bridge amplification.

Pooled amplified cDNA processed in bulk

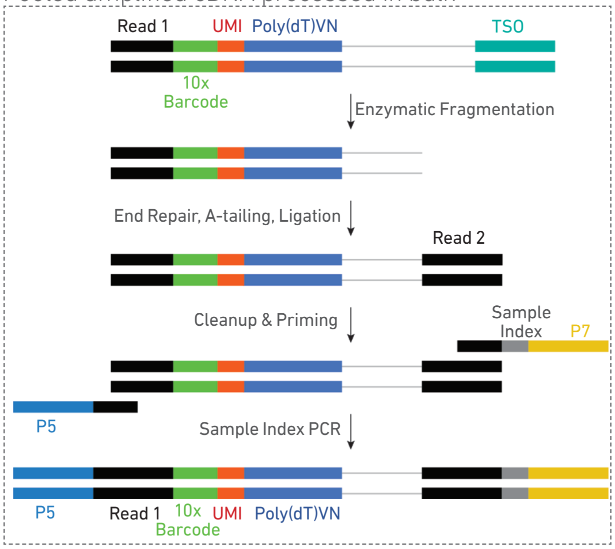

Step 4 Sequencing

A Chromium Single Cell 3’ Gene Expression library comprises standard Illumina paired-end constructs which begin and end with P5 and P7. The 16 bp 10x Barcode and 12 bp UMI are encoded in Read 1, while Read 2 is used to sequence the cDNA fragment. Sample index sequences are incorporated as the i7 index read. TruSeq Read 1 and TruSeq Read 2 are standard Illumina sequencing primer sites used in paired-end sequencing.

Illumina sequencer compatibility, sample indices, library loading and pooling for sequencing are summarized in step 4.

Chromium Single Cell 3' Gene Expression Library

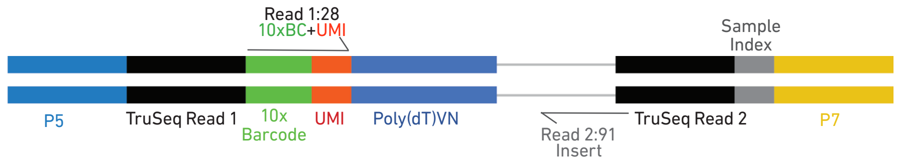

See Appendix for Oligonucleotide Sequences

Tips & Best Practices

TIPS

Icons

TIPS

Tips & Best Practices section includes additional guidance

Signifies critical step requiring accurate execution

Troubleshooting section includes additional guidance

Version Specific Update

VERSION SPECIFIC

Indicates version specific updates in a particular protocol step to inform users who have used a previous version of the product. The updates may be in volume, temperature, calculation instructions etc.

Emulsion-safe Plastics

- Use validated emulsion-safe plastic consumables when handling GEMs as some plastics can destabilize GEMs.

Cell Concentration

- Recommended starting point is to load ~1,600 cells per reaction, resulting in recovery of ~1000 cells, and a multiplet rate of ~0.8%. The optimal input cell concentration is 700-1,200 cells/µl.
- The presence of dead cells in the suspension may also reduce the recovery rate. Consult the 10x Genomics Single Cell Protocols Cell Preparation Guide and the Guidelines for Optimal Sample Preparation flowchart (Documents CG00053 and CG000126 respectively) for more information on preparing cells.

Multiplet Rate (%)	# of Cells Loaded	# of Cells Recovered
~0.4%	~800	~500
~0.8%	~1,600	~1,000
~1.6%	~3,200	~2,000
~2.3%	~4,800	~3,000
~3.1%	~6,400	~4,000
~3.9%	~8,000	~5,000
~4.6%	~9,600	~6,000
~5.4%	~11,200	~7,000
~6.1%	~12,800	~8,000
~6.9%	~14,400	~9,000
~7.6%	~16,000	~10,000

General Reagent Handling

- Fully thaw and thoroughly mix reagents before use.
- Keep all enzymes and Master Mixes on ice during setup and use. Promptly move reagents back to the recommended storage.
- Calculate reagent volumes with 10% excess of 1 reaction values.
- Cover Partitioning Oil tubes and reservoirs to minimize evaporation.
- If using multiple chips, use separate reagent reservoirs for each chip during loading.
- Thoroughly mix samples with the beads during bead-based cleanup steps.

50% Glycerol Solution

- Purchase 50% glycerol solution from Ricca Chemical Company, Glycerin (glycerol), 50% (v/v) Aqueous Solution, PN-3290-32.
- Prepare 50% glycerol solution:

  i. Mix an equal volume of water and 99% Glycerol, Molecular Biology Grade.

  ii. Filter through a 0.2 µm filter.

  iii. Store at –20°C in 1-ml LoBind tubes. 50% glycerol solution should be equilibrated to room temperature before use.

Pipette Calibration

- Follow manufacturer's calibration and maintenance schedules.
- Pipette accuracy is particularly important when using SPRIselect reagents.

Chromium Chip Handling

- Minimize exposure of reagents, chips, and gaskets to sources of particles and fibers, laboratory wipes, frequently opened flip-cap tubes, clothing that sheds fibers, and dusty surfaces.
- Execute steps without pause or delay, unless indicated. When multiple chips are to be used, load, run, and collect the content from one chip before loading the next.
- Fill all unused input wells in rows labeled 1, 2, and 3 on a chip with an appropriate volume of 50% glycerol solution before loading the used wells. DO NOT add glycerol to the Recovery Wells.
- Avoid contacting the bottom surface of the chip with gloved hands and other surfaces. Frictional charging can lead to inadequate priming of the channels, potentially leading to either clogs or wetting failures.
- Minimize the distance that a loaded chip is moved to reach the Chromium Controller.
- Keep the chip horizontal to prevent wetting the gasket with oil, which depletes the input volume and may adversely affect the quality of the resulting emulsion.

10x Chip Holders

- 10x Chip Holders encase Chromium Chips.
- The holder lid flips over to become a stand, holding the chip at 45 degrees for optimal recovery well content removal.
- Squeeze the black sliders on the back side of the holder together to unlock the lid and return the holder to a flat position.

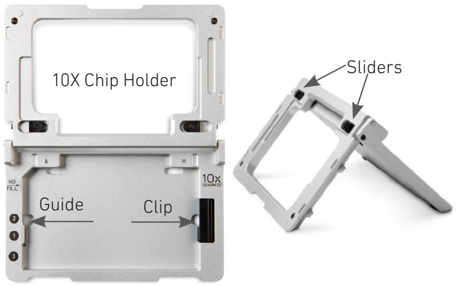

Chromium Chip & Holder Assembly

- Align notch on the chip (upper left corner) and the holder.
- Insert the left-hand side of the chip under the guide. Depress the right-hand side of the chip until the spring-loaded clip engages.
- Close the lid before dispensing reagents into the wells.

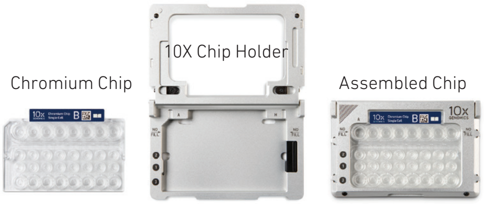

Chromium Chip Loading

- Place the assembled chip and holder flat on the bench with the lid closed.
- Dispense at the bottom of the wells without introducing bubbles.
- When dispensing Gel Beads into the chip, wait for the remainder to drain into the bottom of the pipette tips and dispense again to ensure complete transfer.
- Refer to Load Chromium Chip B for specific instructions.

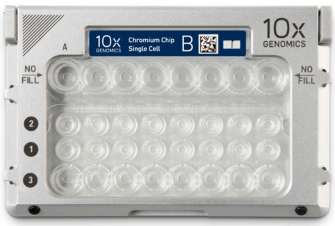

Gel Bead Handling

- Use one tube of Gel Beads per sample. DO NOT puncture the foil seals of tubes not used at the time.
- Equilibrate the Gel Beads strip to room temperature before use.

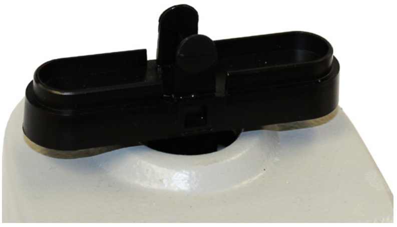

VERSION SPECIFIC

- Store unused Gel Beads at –80°C and avoid more than 12 freeze-thaw cycles. DO NOT store Gel Beads at –20°C.
- Attach a 10x Vortex Adapter to the top of standard laboratory vortexers to vortex the Gel Bead strips.
- After vortexing, remove the Gel Bead strip from the adapter. Flick the Gel Bead strip in a sharp, downward motion maximize Gel Bead recovery. Confirm there are no bubbles at the bottom of the tubes.
- If the required volume of beads cannot be recovered, place the pipette tips against the sidewalls and slowly dispense the Gel Beads back into the tubes. DO NOT introduce bubbles into the tubes and verify that the pipette tips contain no leftover Gel Beads. Withdraw the full volume of beads again by pipetting slowly.

10x Gasket Attachment

- After reagents are loaded, attach the gasket by holding the tongue (curved end, to the right) and hook it on the left-hand tabs of the holder. Gently pull the gasket toward the right and hook it on the two right-hand tabs.
- DO NOT touch the smooth side of the gasket. DO NOT press down on the top of the gasket after attachment.
- Keep the assembly horizontal to avoid wetting the gasket with Partitioning Oil.

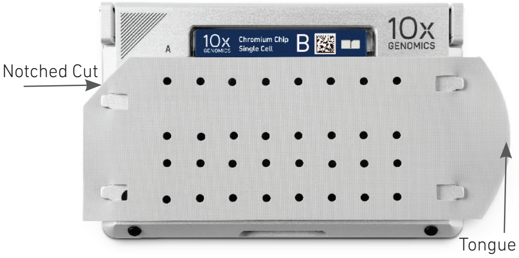

10x Magnetic Separator

- Offers two positions of the magnets (high and low) relative to a tube, depending on its orientation. Flip the magnetic separator over to switch between high (magnet•**High**) or low (magnet•**Low**) positions.
- If using MicroAmp 8-Tube Strips, use the high position (magnet•**High**) only throughout the protocol.

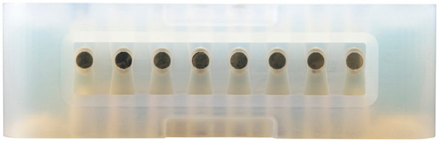

Magnetic Bead Cleanup Steps

- During magnetic bead based cleanup steps that specify waiting "until the solution clears", visually confirm clearing of solution before proceeding to the next step. See adjacent panel for an example.
- The time needed for the solution to clear may vary based on specific step, reagents, volume of reagents etc.

Magnetic beads mixed with reagent

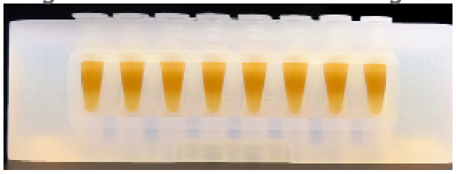

Separation in progress

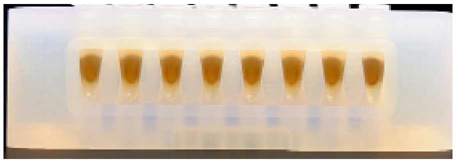

Separation continuing

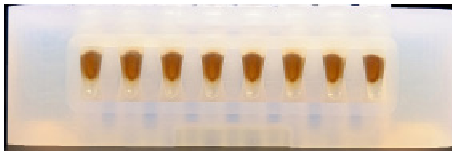

Separation complete; solution is clear

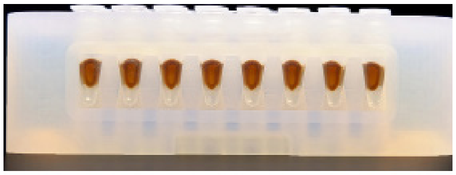

SPRIselect Cleanup & Size Selection

- After aspirating the desired volume of SPRIselect reagent, examine the pipette tips before dispensing to ensure the correct volume is transferred.
- Pipette mix thoroughly as insufficient mixing of sample and SPRIselect reagent will lead to inconsistent results.
- Use fresh preparations of 80% Ethanol.

Tutorial — SPRIselect Reagent:DNA Sample Ratios

SPRI beads selectively bind DNA according to the ratio of SPRIselect reagent (beads).

Example: Ratio = $\frac{\text{Volume of SPRIselect reagent added to the sample}}{\text{Volume of DNA sample}} = \frac{50\ \mu l}{100\ \mu l} = \mathbf{0.5X}$

Schematic of Double Sided Size Selection

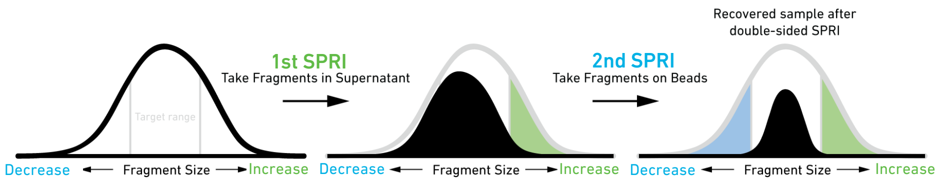

After the first SPRI, supernatant is transferred for a second SPRI while larger fragments are discarded (green). After the second SPRI, fragments on beads are eluted and kept while smaller fragments are discarded (blue). Final sample has a tight fragment size distribution with reduced overall amount (black).

Tutorial — Double Sided Size Selection

Step a – First SPRIselect: Add **50 µl** SPRIselect reagent to **100 µl** sample (**0.5X**).

Ratio = $\frac{\text{Volume of SPRIselect reagent added to the sample}}{\text{Volume of DNA sample}} = \frac{50\ \mu l}{100\ \mu l} = \mathbf{0.5X}$

Step b – Second SPRIselect: Add **30 µl** SPRIselect reagent to supernatant from step a (**0.8X**).

Ratio = $\frac{\text{Total Volume of SPRIselect reagent added to the sample (step a + b)}}{\text{Original Volume of DNA sample}} = \frac{50\ \mu l + 30\ \mu l}{100\ \mu l} = \mathbf{0.8X}$

Enzymatic Fragmentation

- Ensure enzymatic fragmentation reactions are prepared on ice and then loaded into a thermal cycler pre-cooled to 4°C prior to initiating the Fragmentation, End Repair, and A-tailing incubation steps.

Sample Indices in Sample Index PCR

- Choose the appropriate sample index sets to ensure that no sample indices overlap in a multiplexed sequencing run.
- Each well in the i7 Sample Index plate contains a unique mix of 4 oligos.
- The sample indexes can therefore be used in any combination.
- Each sample index set is base-balanced to avoid monochromatic signal issues when it is the sole sample loaded on an Illumina sequencer.

Step 1

GEM Generation & Barcoding

1.1 Prepare Single Cell Master Mix

1.2 Load Chromium Chip B

1.3 Run the Chromium Controller

1.4 Transfer GEMs

1.5 GEM-RT Incubation

1

1.0 GEM Generation & Barcoding

VERSION SPECIFIC

GET STARTED!

Action		Item	10x PN	Preparation & Handling	Storage
**Equilibrate to Room Temperature**		**Chromium Single Cell 3' v3 Gel Beads**	2000059	Equilibrate to room temperature 30 min before loading the chip.	–80°C
	●	**RT Reagent**	2000086	Vortex, verify no precipitate, centrifuge briefly.	–20°C
	●	**Template Switch Oligo**	3000228	Centrifuge briefly, resuspend in 80 µl Low TE Buffer. Vortex 15 sec at maximum speed, centrifuge briefly, leave at room temperature for ≥ 30 min. After resuspension, store at –80°C.	–20°C
	○	**Reducing Agent B**	2000087	Vortex, verify no precipitate, centrifuge briefly.	–20°C
**Place on Ice**	●	**RT Enzyme C**	2000085/ 2000102	Centrifuge briefly before adding to the mix.	–20°C
		**Cell Suspension**			
**Obtain**	●	**Partitioning Oil**	220088	-	Ambient
		**Chromium Chip B Single Cell**	2000060	-	Ambient
		**10x Gasket**	370017/ 3000072	See Tips & Best Practices.	Ambient
		**10x Chip Holder**	330019	See Tips & Best Practices.	Ambient
		**10x Vortex Adapter**	330002	See Tips & Best Practices.	Ambient
		**50% glycerol solution** If using <8 reactions	-	See Tips & Best Practices.	-

Firmware Version 3.16 or higher is required in the Chromium Controller or the Chromium Single Cell Controller used for the Single Cell 3' protocol.

1.1 Prepare Master Mix

a. Prepare Master Mix on ice. Pipette mix 15x and centrifuge briefly.

Master Mix<br>*Add reagents in the order listed*	PN	1X (µl)	4X + 10% (µl)	8X + 10% (µl)
**RT Reagent**	2000086	20.0	88.0	176.0
**Template Switch Oligo**	3000228	3.1	13.9	27.7
**Reducing Agent B**	2000087	2.0	8.7	17.3
**RT Enzyme C**	2000085/<br>2000102	8.3	36.6	73.1
**Total**	-	**33.4**	**147.1**	**294.2**

b. Add **33.4 µl** Master Mix into each tube of a PCR 8-tube strip on ice.

Cell Suspension Volume Calculator Table (for step 1.2)

Volume of Cell Suspension Stock per reaction (µl) | Volume of Nuclease-free Water per reaction (µl)

Cell Stock Concentration (Cells/µl)	Targeted Cell Recovery										
	500	1000	2000	3000	4000	5000	6000	7000	8000	9000	10000
100	8.0<br>38.6	16.0<br>30.6	32.0<br>14.6	n/a	n/a	n/a	n/a	n/a	n/a	n/a	n/a
200	4.0<br>42.6	8.0<br>38.6	16.0<br>30.6	24.0<br>22.6	32.0<br>14.6	40.0<br>6.6	n/a	n/a	n/a	n/a	n/a
300	2.7<br>43.9	5.3<br>41.3	10.7<br>35.9	16.0<br>30.6	21.3<br>25.3	26.7<br>19.9	32.0<br>14.6	37.3<br>9.3	42.7<br>3.9	n/a	n/a
400	2.0<br>44.6	4.0<br>42.6	8.0<br>38.6	12.0<br>34.6	16.0<br>30.6	20.0<br>26.6	24.0<br>22.6	28.0<br>18.6	32.0<br>14.6	36.0<br>10.6	40.0<br>6.6
500	1.6<br>45.0	3.2<br>43.4	6.4<br>40.2	9.6<br>37.0	12.8<br>33.8	16.0<br>30.6	19.2<br>27.4	22.4<br>24.2	25.6<br>21.0	28.8<br>17.8	32.0<br>14.6
600	1.3<br>45.3	2.7<br>43.9	5.3<br>41.3	8.0<br>38.6	10.7<br>35.9	13.3<br>33.3	16.0<br>30.6	18.7<br>27.9	21.3<br>25.3	24.0<br>22.6	26.7<br>19.9
700	1.1<br>45.5	2.3<br>44.3	4.6<br>42.0	6.9<br>39.7	9.1<br>37.5	11.4<br>35.2	13.7<br>32.9	16.0<br>30.6	18.3<br>28.3	20.6<br>26.0	22.9<br>23.7
800	1.0<br>45.6	2.0<br>44.6	4.0<br>42.6	6.0<br>40.6	8.0<br>38.6	10.0<br>36.6	12.0<br>34.6	14.0<br>32.6	16.0<br>30.6	18.0<br>28.6	20.0<br>26.6
900	0.9<br>45.7	1.8<br>44.8	3.6<br>43.0	5.3<br>41.3	7.1<br>39.5	8.9<br>37.7	10.7<br>35.9	12.4<br>34.2	14.2<br>32.4	16.0<br>30.6	17.8<br>28.8
1000	0.8<br>45.8	1.6<br>45.0	3.2<br>43.4	4.8<br>41.8	6.4<br>40.2	8.0<br>38.6	9.6<br>37.0	11.2<br>35.4	12.8<br>33.8	14.4<br>32.2	16.0<br>30.6
1100	0.7<br>45.9	1.5<br>45.1	2.9<br>43.7	4.4<br>42.2	5.8<br>40.8	7.3<br>39.3	8.7<br>37.9	10.2<br>36.4	11.6<br>35.0	13.1<br>33.5	14.5<br>32.1
1200	0.7<br>45.9	1.3<br>45.3	2.7<br>43.9	4.0<br>42.6	5.3<br>41.3	6.7<br>39.9	8.0<br>38.6	9.3<br>37.3	10.7<br>35.9	12.0<br>34.6	13.3<br>33.3
1300	0.6<br>46.0	1.2<br>45.4	2.5<br>44.1	3.7<br>42.9	4.9<br>41.7	6.2<br>40.4	7.4<br>39.2	8.6<br>38.0	9.8<br>36.8	11.1<br>35.5	12.3<br>34.3
1400	0.6<br>46.0	1.1<br>45.5	2.3<br>44.3	3.4<br>43.2	4.6<br>42.0	5.7<br>40.9	6.9<br>39.7	8.0<br>38.6	9.1<br>37.5	10.3<br>36.3	11.4<br>35.2
1500	0.5<br>46.1	1.1<br>45.5	2.1<br>44.5	3.2<br>43.4	4.3<br>42.3	5.3<br>41.3	6.4<br>40.2	7.5<br>39.1	8.5<br>38.1	9.6<br>37.0	10.7<br>35.9
1600	0.5<br>46.1	1.0<br>45.6	2.0<br>44.6	3.0<br>43.6	4.0<br>42.6	5.0<br>41.6	6.0<br>40.6	7.0<br>39.6	8.0<br>38.6	9.0<br>37.6	10.0<br>36.6
1700	0.5<br>46.1	0.9<br>45.7	1.9<br>44.7	2.8<br>43.8	3.8<br>42.8	4.7<br>41.9	5.6<br>41.0	6.6<br>40.0	7.5<br>39.1	8.5<br>38.1	9.4<br>37.2
1800	0.4<br>46.2	0.9<br>45.7	1.8<br>44.8	2.7<br>43.9	3.6<br>43.0	4.4<br>42.2	5.3<br>41.3	6.2<br>40.4	7.1<br>39.5	8.0<br>38.6	8.9<br>37.7
1900	0.4<br>46.2	0.8<br>45.8	1.7<br>44.9	2.5<br>44.1	3.4<br>43.2	4.2<br>42.4	5.1<br>41.5	5.9<br>40.7	6.7<br>39.9	7.6<br>39.0	8.4<br>38.2
2000	0.4<br>46.2	0.8<br>45.8	1.6<br>45.0	2.4<br>44.2	3.2<br>43.4	4.0<br>42.6	4.8<br>41.8	5.6<br>41.0	6.4<br>40.2	7.2<br>39.4	8.0<br>38.6

Grey boxes: Volumes that would exceed the allowable water volume in each reaction

Yellow boxes: Indicate a low transfer volume that may result in higher cell load variability

Blue boxes: Optimal range of cell stock concentration to maximize the likelihood of achieving the desired cell recovery target

1.2 Load Chromium Chip B

See Tips & Best Practices for chip handling instructions. When loading the chip, raising and depressing the pipette plunger should each take ~**5 sec**. When dispensing, raise the pipette tips at the same rate as the liquid is rising, keeping the tips slightly submerged.

TIPS

a. Assemble Chromium Chip B in a 10x Chip Holder. See Tips & Best Practices.

VERSION SPECIFIC

b. Dispense 50% Glycerol Solution into Unused Chip Wells (if < 8 samples per chip)

i. **75 µl** to unused wells in **row labeled 1**.
ii. **40 µl** to unused wells in **row labeled 2**.
iii. **280 µl** to unused wells in **row labeled 3**.

DO NOT add 50% glycerol solution to the top row of Recovery Wells. DO NOT use any substitute for 50% glycerol solution.

c. Prepare Master Mix + Cell Suspension
Refer to the Cell Suspension Volume Calculator Table and add the appropriate volume of nuclease-free water and corresponding volume of single cell suspension to Master Mix for a total of **80 µl** in each tube. Gently pipette mix the single cell suspension before adding to the Master Mix.

VERSION SPECIFIC

d. Load Row Labeled 1
Gently pipette mix the Master Mix + Cell Suspension and using the same pipette tip, dispense **75 µl** Master Mix + Cell Suspension into the bottom center of each well in **row labeled 1** without introducing bubbles.

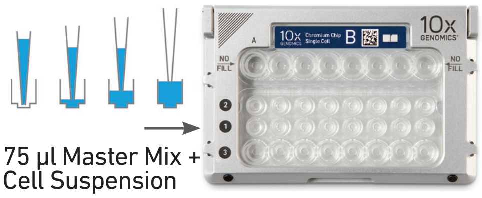

e. Prepare Gel Beads
Snap the Gel Bead strip into a 10x Vortex Adapter. Vortex **30 sec.** Remove the Gel Bead strip and flick in a sharp, downward motion to ensure maximum recovery. Confirm there are no bubbles at the bottom of the tubes and liquid levels look even.

f. Load Row Labeled 2
Puncture the foil seal of the Gel Bead tubes. Slowly aspirate **40 µl** Gel Beads. Dispense into the wells in **row labeled 2** without introducing bubbles.

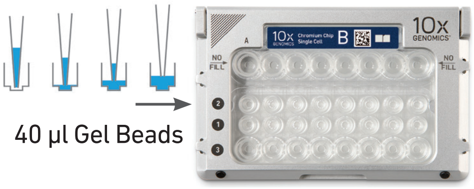

VERSION SPECIFIC

g. Load Row Labeled 3
Dispense **280 µl** Partitioning Oil into the wells in **row labeled 3** by pipetting two aliquots of **140 µl** from a reagent reservoir. Failure to add Partitioning Oil can damage the Chromium Controller.

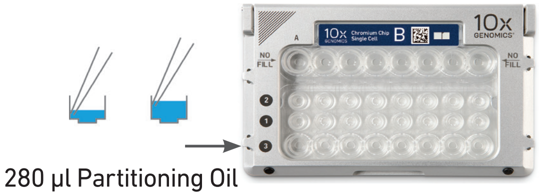

h. Attach 10x Gasket
Align the notch with the top left-hand corner. Ensure the gasket holes are aligned with the wells. Avoid touching the smooth gasket surface. DO NOT press down on the gasket.

Keep horizontal to avoid wetting the gasket.

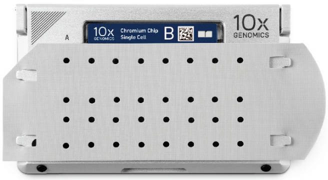

1.3 Run the Chromium Controller

a. Press the eject button on the Controller to eject the tray.

b. Place the assembled chip with the gasket in the tray. Press the button to retract the tray.

c. Confirm the Chromium Single Cell B program on screen. Press the play button.

d. At completion of the run **(~8.5 min)**, the Controller will chime. **Immediately** proceed to the next step.

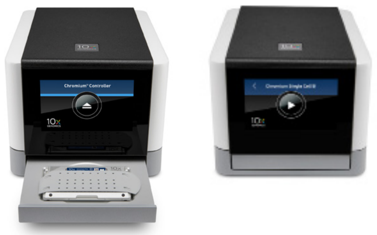

1.4 Transfer GEMs

a. Place a tube strip on ice.

b. Press the eject button of the Controller and remove the chip.

c. Discard the gasket. Open the chip holder. Fold the lid back until it clicks to expose the wells at 45 degrees.
Ensure that the partitioning oil from the wells does not spill when exposing the wells.

d. Check the volume in rows 1-3. Abnormally high volume in any well indicates a clog.

e. Slowly aspirate **100 µl** GEMs from the lowest points of the Recovery Wells in the top row without creating a seal between the tips and the bottom of the wells.

f. Withdraw pipette tips from the wells. GEMs should appear opaque and uniform across all channels. Excess Partitioning Oil (clear) in the pipette tips indicates a potential clog.

g. Over the course of ~**20 sec**, dispense GEMs into the tube strip on ice with the pipette tips against the sidewalls of the tubes.

h. If multiple chips are run back-to-back, cap/ cover the GEM-containing tube strip and place on ice for no more than **1 h**.

Expose Wells at 45 Degrees

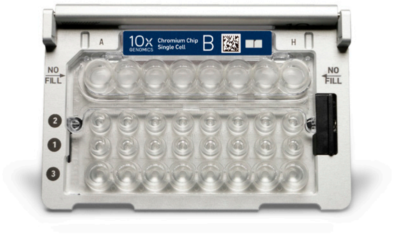

Transfer GEMs

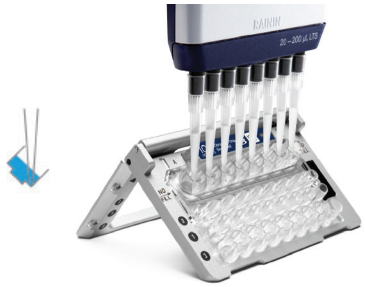

GEMs

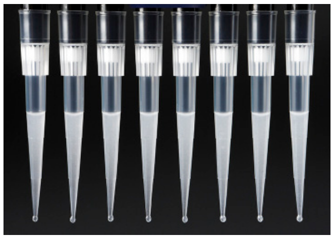

1.5 GEM-RT Incubation

Use a thermal cycler that can accommodate at least 100 µl volume. A volume of 125 µl is the preferred setting on Bio-Rad C1000 Touch. In alternate thermal cyclers, use highest reaction volume setting.

a. Incubate in a thermal cycler with the following protocol.

Lid Temperature	Reaction Volume	Run Time
53°C	125 µl	~55 min
**Step**	**Temperature**	**Time**
1	53°C	00:45:00
2	85°C	00:05:00
3	4°C	Hold

STOP

b. Store at **4°C** for up to **72 h** or at **–20°C** for up to a **week**, or proceed to the next step.

Step 2

Post GEM–RT Cleanup & cDNA Amplification

2.1 Post GEM–RT Cleanup – Dynabeads

2.2 cDNA Amplification

2.3 cDNA Cleanup – SPRIselect

2.4 cDNA QC & Quantification

2

2.0
Post GEM-RT Cleanup & cDNA Amplification

GET STARTED!

Action	Item	10x PN	Preparation & Handling	Storage
**Equilibrate to Room Temperature**	**Reducing Agent B**	2000087	Thaw, vortex, verify no precipitate, centrifuge.	−20°C
	**cDNA Primers**	2000089	Vortex, centrifuge briefly.	−20°C
	**Beckman Coulter SPRIselect Reagent**	-	Manufacturer's recommendations.	-
	**Agilent Bioanalyzer High Sensitivity Kit** If used for QC and quantification	-	Manufacturer's recommendations.	-
	**Agilent TapeStation ScreenTape and Reagents** If used for QC and quantification	-	Manufacturer's recommendations.	-
	**Qubit dsDNA HS Assay Kit** If used for QC and quantification	-	Manufacturer's recommendations.	-
	**Dynabeads MyOne SILANE**	2000048	Vortex thoroughly (≥30 sec) **immediately** before adding to the mix.	4°C
**Place on ice**	**Amp Mix**	2000047/ 2000103	Vortex, centrifuge briefly.	−20°C
**Thaw at 65°C**	**Cleanup Buffer**	2000088	Thaw for 10 min at 65°C at max speed on a thermomixer. Verify no visible crystals. Cool to room temperature.	−20°C
**Obtain**	**Recovery Agent**	220016	-	Ambient
	**Qiagen Buffer EB**	-	Manufacturer's recommendations.	-
	**Bio-Rad 10% Tween 20**	-	Manufacturer's recommendations.	-
	**10x Magnetic Separator**	230003	-	Ambient
	**Prepare 80% Ethanol** Prepare 15 ml for 8 reactions.	-	-	-

2.1 Post GEM-RT Cleanup – Dynabeads

a. Add **125 µl** Recovery Agent to each sample at room temperature. DO NOT pipette mix or vortex the biphasic mixture. Wait **60 sec**.

The resulting biphasic mixture contains Recovery Agent/Partitioning Oil (pink) and aqueous phase (clear), with no persisting emulsion (opaque).
If biphasic separation is incomplete, cap the tube strip, centrifuge briefly, and proceed to step b.

Biphasic Mixture

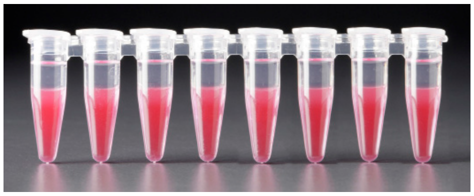

A smaller aqueous phase volume indicates a clog during GEM generation.

b. Slowly remove **125 µl** Recovery Agent/ Partitioning Oil (pink) from the bottom of the tube. DO NOT aspirate any aqueous sample.

Remove Recovery Agent

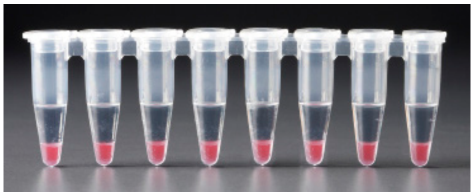

c. Prepare Dynabeads Cleanup Mix.

Dynabeads Cleanup Mix<br>*Add reagents in the order listed*	PN	1X (µl)	4X + 10% (µl)	8X + 10% (µl)
**Cleanup Buffer**	2000088	182	801	1602
**Dynabeads MyOne SILANE**<br>Vortex thoroughly (≥**30 sec**) **immediately** before adding to the mix. If still clumpy, pipette mix to resuspend completely.<br>DO NOT centrifuge before use.	2000048	8	35	70
**Reducing Agent B**	2000087	5	22	44
**Nuclease-free Water**		5	22	44

VERSION SPECIFIC

Add Dynabeads Cleanup Mix

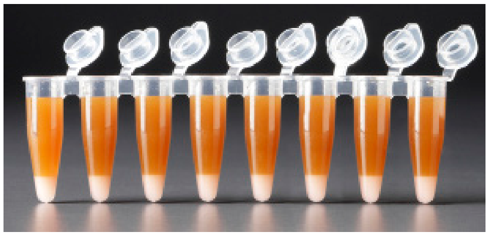

d. Vortex and add **200 µl** to each sample. Pipette mix 10x (pipette set to 200 µl).

e. Incubate **10 min** at **room temperature**. Pipette mix again at ~**5 min** after start of incubation to resuspend settled beads.

f. Prepare Elution Solution I. Vortex and centrifuge briefly.

**Elution Solution I** *Add reagents in the order listed*	PN	1X (µl)	10X (µl)
**Buffer EB**	-	98	980
**10% Tween 20**	-	1	10
◯ **Reducing Agent B**	2000087	1	10
**Total**	**-**	**100**	**1000**

TIPS

g. At the end of **10 min** incubation, place on a 10x Magnetic Separator•**High** position (magnet•**High**) until the solution clears.

A white interface between the aqueous phase and Recovery Agent is normal.

h. Remove the supernatant.

i. Add **300 µl** 80% ethanol to the pellet while on the magnet. Wait **30 sec**.

j. Remove the ethanol.

k. Add **200 µl** 80% ethanol to pellet. Wait **30 sec**.

l. Remove the ethanol.

m. Centrifuge briefly. Place on the magnet•**Low**.

n. Remove remaining ethanol. Air dry for **1 min**.

o. Remove from the magnet. Immediately add **35.5 µl** Elution Solution I.

p. Pipette mix (pipette set to 30 µl) without introducing bubbles.

q. Incubate **2 min** at **room temperature**.

r. Place on the magnet•**Low** until the solution clears.

s. Transfer **35 µl** sample to a new tube strip.

2.2 cDNA Amplification

a. Prepare cDNA Amplification Mix on ice. Vortex and centrifuge briefly.

**cDNA Amplification Reaction Mix** *Add reagents in the order listed*	PN	1X (µl)	4X + 10% (µl)	8X + 10% (µl)
○ **Amp Mix**	2000047/ 2000103	50	220	440
● **cDNA Primers**	2000089	15	66	132
**Total**	-	**65**	**286**	**572**

b. Add **65 µl** cDNA Amplification Reaction Mix to **35 µl** sample.

c. Pipette mix 15x (pipette set to 90 µl). Centrifuge briefly.

d. Incubate in a thermal cycler with the following protocol.

Lid Temperature	Reaction Volume	Run Time
105°C	100 µl	~30-45 min
**Step**	**Temperature**	**Time**
1	98°C	00:03:00
2	98°C	00:00:15
3	VERSION SPECIFIC 63°C Version Specific Updated Temperature	00:00:20
4	72°C	00:01:00
5	Go to Step 2, see table below for total # of cycles	
6	72°C	00:01:00
7	4°C	Hold

The optimal number of cycles is a trade-off between generating sufficient final mass for library construction and minimizing PCR amplification artifacts. The number of cDNA cycles should also be reduced if large numbers of cells are sampled.

Recommended starting point for cycle number optimization.

Cell Load	Total Cycles
<500	13
500–6,000	12
>6,000	11

STOP

e. Store at **4°C** for up to **72 h** or proceed to the next step.

2.3 cDNA Cleanup – SPRIselect

a. Vortex to resuspend the SPRIselect reagent. Add **60 µl** SPRIselect reagent **(0.6X)** to each sample and pipette mix 15x (pipette set to 150 µl).

b. Incubate **5 min** at **room temperature**.

c. Place on the magnet•**High** until the solution clears.

d. Remove the supernatant.

e. Add **200 µl** 80% ethanol to the pellet. Wait **30 sec**.

f. Remove the ethanol.

g. **Repeat** steps e and f for a total of 2 washes.

h. Centrifuge briefly and place on the magnet•**Low**.

i. Remove any remaining ethanol. Air dry for **2 min**. DO NOT exceed **2 min** as this will decrease elution efficiency.

j. Remove from the magnet. Add **40.5 µl** Buffer EB. Pipette mix 15x.

k. Incubate **2 min** at **room temperature**.

l. Place the tube strip on the magnet•**High** until the solution clears.

m. Transfer **40 µl** sample to a new tube strip.

STOP **n.** Store at **4°C** for up to **72 h** or at **–20°C** for up to **4 weeks**, or proceed to the next step.

2.4 cDNA QC & Quantification

a. Run **1 µl** of sample (Dilution Factor 1:10) on an Agilent Bioanalyzer High Sensitivity chip.
For input cells with low RNA content (<1pg total RNA/cell), 1 µl undiluted product may be run.
Lower molecular weight product (35 – 150 bp) may be present. This is normal and does not affect sequencing or application performance.

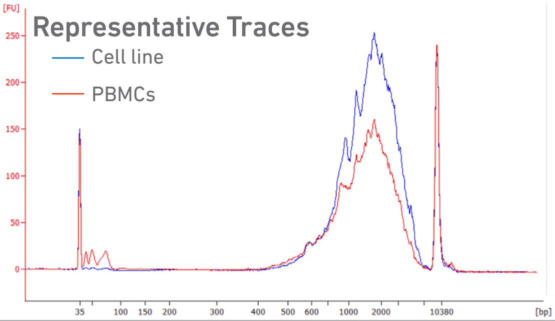

EXAMPLE CALCULATION

i. Select Region

Under the “Electropherogram” view choose the “Region Table”. Manually select the region of ~200 – ~9000 bp

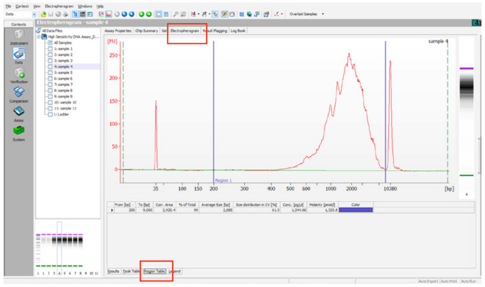

ii. Note Concentration [pg/µl]

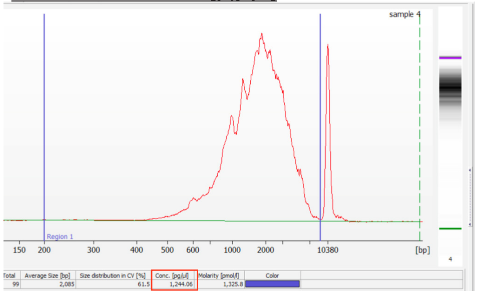

iii. Calculate

Multiply the cDNA concentration [pg/µl] reported via the Agilent 2100 Expert Software by the elution volume (40 µl) of the Post cDNA Amplification Reaction Clean Up sample (taking any dilution factors into account) and then divide by 1000 to obtain the total cDNA yield in ng.

Example Calculation of cDNA Total Yield

Concentration: 1244.06 pg/µl
Elution Volume: 40
Dilution Factor: 10

Total cDNA Yield

= Conc’n (pg/µl) x Elution Volume (µl) x Dilution Factor / 1000 (pg/ng)

= 1244.06 (pg/µl) x 40 (µl) x 10 / 1000 (pg/ng) = 497.62 ng

VERSION SPECIFIC

Carry forward **ONLY 25%** of total cDNA yield into 3' Gene Expression Library Construction (step 3)

= 0.25 x Total cDNA yield

= 0.25 x 497.62= 124.4ng

Refer to step 3.5 for appropriate number of Sample Index PCR cycles based on carry forward cDNA yield/input cDNA.

Alternate Quantification Methods:

- Agilent TapeStation. See Appendix for representative traces

Agilent Bioanalyzer or Agilent TapeStaion are the recommended methods for accurate quantification.

(If using Qubit Fluorometer and Qubit dsDNA HS Assay Kit, see Appendix)

Step 3

3' Gene Expression Library Construction

3.1 Fragmentation, End Repair & A-tailing
3.2 Post Fragmentation End Repair & A-tailing Double Sided Size Selection – SPRIselect
3.3 Adaptor Ligation
3.4 Post Ligation Cleanup – SPRIselect
3.5 Sample Index PCR
3.6 Post Sample Index PCR Double Sided Size Selection – SPRIselect
3.7 Post Library Construction QC

3.0
3' Gene Expression Library Construction

GET STARTED!

Action	Item	10x PN	Preparation & Handling	Storage
**Equilibrate to Room Temperature**	**Fragmentation Buffer**	2000091	Vortex, verify no precipitate, centrifuge briefly.	−20°C
	**Adaptor Oligos**	2000094	Vortex, centrifuge briefly.	−20°C
	**Ligation Buffer**	2000092	Vortex, verify no precipitate, centrifuge briefly.	−20°C
	**SI Primer**	2000095	-	−20°C
	**Chromium i7 Sample Index Plate**	220103	-	−20°C
	**Beckman Coulter SPRIselect Reagent**	-	Manufacturer's recommendations.	-
	**Agilent TapeStation Screen Tape and Reagents** If used for QC		Manufacturer's recommendations.	
	**Agilent Bioanalyzer High Sensitivity kit**	-	Manufacturer's recommendations.	-
**Place on Ice**	**Fragmentation Enzyme**	2000090/ 2000104	Centrifuge briefly.	−20°C
	**DNA Ligase**	220110/ 220131	Centrifuge briefly.	−20°C
	**Amp Mix**	2000047/ 2000103	Centrifuge briefly.	−20°C
	**KAPA Library Quantification Kit for Illumina Platforms**	-	Manufacturer's recommendations.	-
**Obtain**	**Qiagen Buffer EB**	-	-	Ambient
	**10x Magnetic Separator**	230003	See Tips & Best Practices.	Ambient
	**Prepare 80% Ethanol** Prepare 20 ml for 8 reactions	-	Prepare fresh.	Ambient

3.1 Fragmentation, End Repair & A-tailing

a. Prepare a thermal cycler with the following incubation protocol.

Lid Temperature	Reaction Volume	Run Time
65°C	50 µl	~35 min
**Step**	**Temperature**	**Time**
Pre-cool block<br>Pre-cool block prior to preparing the Fragmentation Mix	4°C	Hold
Fragmentation	32°C	00:05:00
End Repair & A-tailing	65°C	00:30:00
Hold	4°C	Hold

b. Vortex Fragmentation Buffer. Verify there is no precipitate.

c. Prepare Fragmentation Mix on ice. Pipette mix and centrifuge briefly.

Fragmentation Mix<br>*Add reagents in the order listed*	PN	1X (µl)	4X + 10% (µl)	8X + 10% (µl)
**Fragmentation Buffer**	2000091/ 2000104	5	22	44
**Fragmentation Enzyme**	2000090	10	44	88
**Total**	-	**15**	**66**	**132**

VERSION SPECIFIC

d. Transfer **ONLY 10 µl** purified cDNA sample from cDNA Cleanup (step 2.3n) to a tube strip.

Note that only **10 µl** (25%) cDNA sample is sufficient for generating 3' Gene Expression library. The remaining **30 µl** (75%) cDNA sample can be stored at **4°C** for up to **72 h** or at **–20°C** for up to **4 weeks** for generating additional 3' Gene Expression libraries.

e. Add **25 µl** Buffer EB to each sample.

f. Add **15 µl** Fragmentation Mix to each sample.

g. Pipette mix 15x (pipette set to 35 µl) on ice. Centrifuge briefly.

h. Transfer into the pre-cooled thermal cycler **(4°C)** and press “SKIP” to initiate the protocol.

3.2 Post Fragmentation, End Repair & A-tailing Double Sided Size Selection – SPRIselect

a. Vortex to resuspend SPRIselect reagent. Add **30 µl** SPRIselect **(0.6X)** reagent to each sample. Pipette mix 15x (pipette set to 75 µl).

b. Incubate **5 min** at **room temperature**.

c. Place on the magnet•**High** until the solution clears. DO NOT discard supernatant.

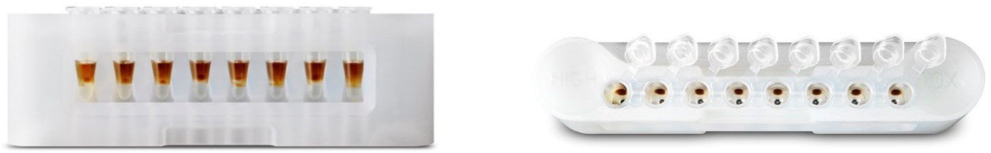

d. Transfer 75 **µl** supernatant to a new tube strip.

e. Vortex to resuspend SPRIselect reagent. Add **10 µl** SPRIselect reagent **(0.8X)** to each sample. Pipette mix 15x (pipette set to 80 µl).

f. Incubate **5 min** at **room temperature**.

g. Place on the magnet•**High** until the solution clears.

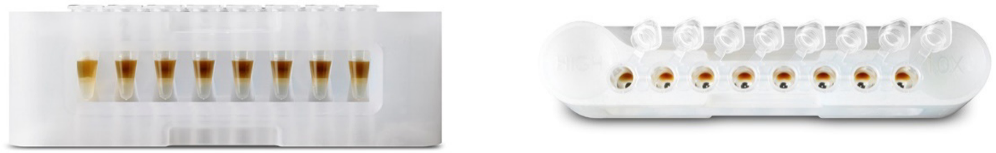

h. Remove **80 µl** supernatant. DO NOT discard any beads.

i. Add **125 µl** 80% ethanol to the pellet. Wait **30 sec.**

j. Remove the ethanol.

k. **Repeat** steps i and j for a total of 2 washes.

l. Centrifuge briefly. Place on the magnet•**Low** until the solution clears. Remove remaining ethanol. DO NOT over dry to ensure maximum elution efficiency.

m. Remove from the magnet. Add **50.5 µl** Buffer EB to each sample. Pipette mix 15x.

n. Incubate **2 min** at **room temperature**.

o. Place on the magnet•**High** until the solution clears.

p. Transfer **50 µl** sample to a new tube strip.

3.3 Adaptor Ligation

a. Prepare Adaptor Ligation Mix. Pipette mix and centrifuge briefly.

Adaptor Ligation Mix<br>*Add reagents in the order listed*	PN	1X (μl)	4X + 10% (μl)	8X + 10% (μl)
**Ligation Buffer**	2000092	20	88	176
**DNA Ligase**	220110/ 220131	10	44	88
**Adaptor Oligos**	2000094	20	88	176
**Total**	-	**50**	**220**	**440**

b. Add **50 μl** Adaptor Ligation Mix to **50 μl** sample. Pipette mix 15x (pipette set to 90 μl). Centrifuge briefly.

c. Incubate in a thermal cycler with the following protocol.

Lid Temperature	Reaction Volume	Run Time
30°C	100 μl	15 min
**Step**	**Temperature**	**Time**
1	20°C	00:15:00
2	4°C	Hold

3.4
Post Ligation Cleanup – SPRIselect

a. Vortex to resuspend SPRIselect Reagent. Add **80 µl** SPRIselect Reagent (**0.8X**) to each sample. Pipette mix 15x (pipette set to 150 µl).

b. Incubate **5 min** at **room temperature**.

c. Place on the magnet•**High** until the solution clears.

d. Remove the supernatant.

e. Add **200 µl** 80% ethanol to the pellet. Wait **30 sec**.

f. Remove the ethanol.

g. **Repeat** steps e and f for a total of 2 washes.

h. Centrifuge briefly. Place on the magnet•**Low**.

i. Remove any remaining ethanol. Air dry for **2 min**. DO NOT exceed **2 min** as this will decrease elution efficiency.

j. Remove from the magnet. Add **30.5 µl** Buffer EB. Pipette mix 15x.

k. Incubate **2 min** at **room temperature**.

l. Place on the magnet•**Low** until the solution clears.

m. Transfer **30 µl** sample to a new tube strip.

3.5 Sample Index PCR

a. Choose the appropriate sample index sets to ensure that no sample indices overlap in a multiplexed sequencing run. Record the 10x Sample Index name (PN-220103 Chromium i7 Sample Index Plate well ID) used.

b. Prepare Sample Index PCR Mix.

Sample Index PCR Mix *Add reagents in the order listed*	PN	1X (µl)	4X + 10% (µl)	8X + 10% (µl)
Amp Mix	2000047/ 2000103	50	220	440
SI Primer	2000095	10	44	88
Total	-	**60**	**264**	**528**

c. Add **60 µl** Sample Index PCR Mix to **30 µl** sample.

d. Add **10 µl** of an individual Chromium i7 Sample Index to each well and record the well ID used. Pipette mix 5x (pipette set to 90 µl). Centrifuge briefly.

e. Incubate in a thermal cycler with the following protocol.

Lid Temperature	Reaction Volume	Run Time
105°C	100 µl	~25-40 min
**Step**	**Temperature**	**Time**
1	98°C	00:00:45
2	98°C	00:00:20
3	54°C	00:00:30
4	72°C	00:00:20
5	Go to step 2, see below for # of cycles	
6	72°C	00:01:00
7	4°C	Hold

The total cycles should be optimized based on 25% carry forward cDNA yield/input calculated during Post cDNA Amplification QC & Quantification (step 2.4)

Recommended cycle numbers

cDNA Input	Total Cycles
1-25 ng	14-16
25-150 ng	12-14
150-500 ng	10-12
500-1,000 ng	8-10
1,000-1,500 ng	6-8

STOP

f. Store at **4°C** for up to **72 h** or proceed to the next step.

3.6 Post Sample Index PCR Double Sided Size Selection – SPRIselect

a. Vortex to resuspend the SPRIselect reagent. Add **60 µl** SPRIselect Reagent **(0.6X)** to each sample. Pipette mix 15x (pipette set to 150 µl).

b. Incubate **5 min** at **room temperature**.

c. Place the magnet•**High** until the solution clears. DO NOT discard supernatant.

d. Transfer **150 µl** supernatant to a new tube strip.

e. Vortex to resuspend the SPRIselect reagent. Add **20 µl** SPRIselect Reagent **(0.8X)** to each sample. Pipette mix 15x (pipette set to 150 µl).

f. Incubate **5 min** at **room temperature**.

g. Place the magnet•**High** until the solution clears.

h. Remove **165 µl** supernatant. DO NOT discard any beads.

i. With the tube still in the magnet, add **200 µl** 80% ethanol to the pellet. Wait **30 sec**.

j. Remove the ethanol.

k. **Repeat** steps i and j for a total of 2 washes.

l. Centrifuge briefly. Place on the magnet•**Low**. Remove remaining ethanol.

m. Remove from the magnet. Add **35.5 µl** Buffer EB. Pipette mix 15x.

n. Incubate **2 min** at **room temperature**.

o. Place on the magnet•**Low** until the solution clears.

p. Transfer **35 µl** to a new tube strip.

STOP

q. Store at **4°C** for up to **72 h** or at **–20°C** for **long-term** storage.

3.7 Post Library Construction QC

Run **1 µl** sample at **1:10 dilution** on an Agilent Bioanalyzer High Sensitivity chip.

Representative Trace

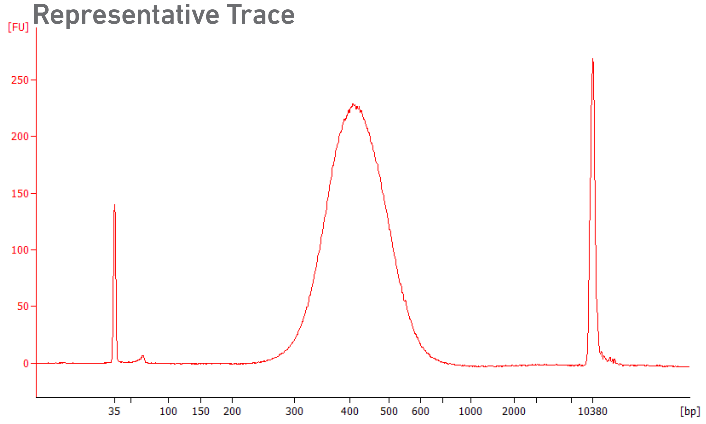

Determine the average fragment size from the Bioanalyzer trace. This will be used as the insert size for library quantification.

Alternate QC Method:

- Agilent TapeStation. See Appendix for representative traces

See Appendix for Post Library Construction Quantification

Sequencing

4

Sequencing Libraries

Single Cell 3' Gene Expression libraries comprise standard Illumina paired-end constructs which begin with P5 and end with P7. 16 bp 10x Barcodes are encoded at the start of TruSeq Read 1, while 8 bp sample index sequences are incorporated as the i7 index read. TruSeq Read 1 and Read 2 are standard Illumina sequencing primer sites used in paired-end sequencing. TruSeq Read 1 is used to sequence 16 bp 10x Barcodes and 12 bp UMI. Sequencing these libraries produce a standard Illumina BCL data output folder.

Chromium Single Cell 3' Gene Expression Library

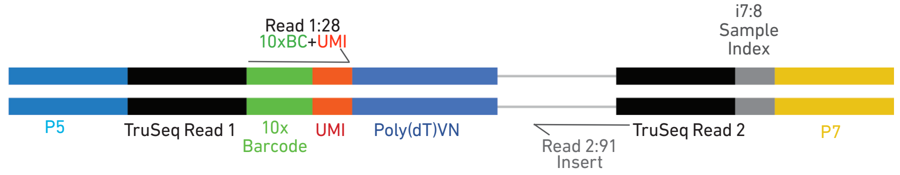

Illumina Sequencer Compatibility

The compatibility of the listed sequencers has been verified by 10x Genomics. Some variation in assay performance is expected based on sequencer choice. For more information about performance variation, visit the 10x Genomics Support website.

- MiSeq
- NextSeq 500/550*
- HiSeq 2500 (Rapid Run)
- HiSeq 3000/4000
- NovaSeq

*Sequencing Chromium Single Cell libraries on the NextSeq 500/550 platform may yield reduced sequence quality and sensitivity relative to the MiSeq, HiSeq, and NovaSeq platforms. Refer to the 10x Genomics Support website for more information.

Sample Indices

Each sample index in the Chromium i7 Sample Index Kit (PN-120262) is a mix of 4 different sequences to balance across all 4 nucleotides. If multiple samples are pooled in a sequence lane, the sample index name (i.e. the Chromium i7 Sample Index plate well ID, SI-GA-) is needed in the sample sheet used for generating FASTQs with “cellranger mkfastq”. Samples utilizing the same sample index should not be pooled together, or run on the same flow cell lane, as this would not enable correct sample demultiplexing.

3' Gene Expression Library Sequencing Depth & Run Parameters

**Sequencing Depth**	Minimum 20,000 read pairs per cell
**Sequencing Type**	Paired-end, single indexing
**Sequencing Read**	Recommended Number of Cycles
Read 1	28 cycles
i7 Index	8 cycles
i5 Index	0 cycles
Read 2	91 cycles

Library Loading

Once quantified and normalized, the 3' Gene Expression libraries should be denatured and diluted as recommended for Illumina sequencing platforms. Refer to Illumina documentation for denaturing and diluting libraries. Refer to the 10x Genomics Support website, for more information.

Instrument	Loading Concentration (pM)	PhiX (%)
MiSeq	11	1
NextSeq 500	1.8	1
HiSeq 2500 (RR)	11	1
HiSeq 4000	240	1
NovaSeq	150*/300	1

* Use 150 pM loading concentration for Illumina XP workflow.

Library Pooling

The 3' Gene Expression libraries maybe pooled for sequencing, taking into account the differences in cell number and per-cell read depth requirements between each library. Samples utilizing the same sample index should not be pooled together, or run on the same flow cell lane, as this would not enable correct sample demultiplexing.

Troubleshooting

5

5.1 GEMs

STEP	NORMAL	REAGENT CLOGS & WETTING FAILURES
1.4 d<br>After Chip B is removed from the Controller and the wells are exposed	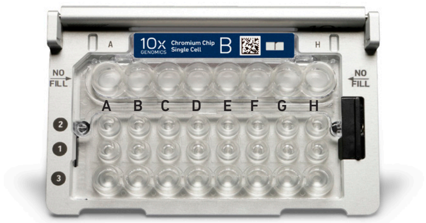<br>All 8 Recovery Wells are similar in volume and opacity.	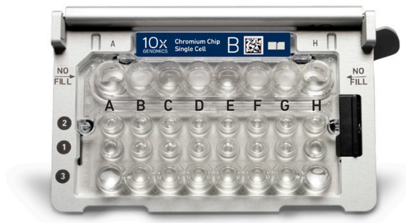<br>Recovery Well G indicates a reagent clog. Recovery Well C and E indicate a wetting failure. Recovery Wells B, D, and F are normal. Wells A and H contain 50% Glycerol Solution.
		The image indicates clogs in the Gel Bead line (orange arrow) and the sample line (yellow arrow) as evidenced by higher than usual volumes in the input wells.<br>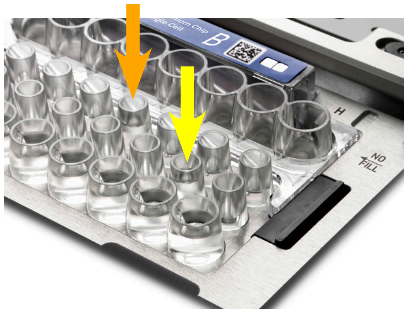
1.4 f<br>Transfer GEMs from Chip E Recovery Wells	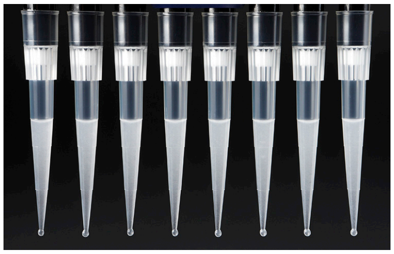<br>All liquid levels are similar in volume and opacity without air trapped in the pipette tips.	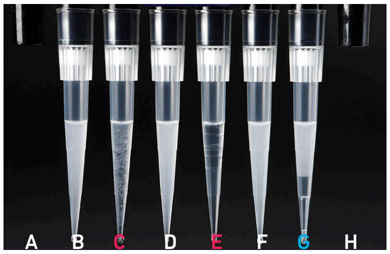<br>Pipette tips C and E indicate a wetting failure. Pipette tip C contains partially emulsified GEMs. Emulsion is absent in pipette tip E. Pipette tip G indicates a reagent clog.

STEP	NORMAL	REAGENT CLOGS & WETTING FAILURES
2.1 a<br>After transfer of the GEMs + Recovery Agent	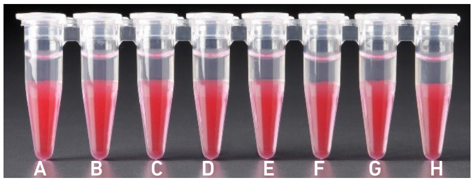<br>All liquid levels are similar in the aqueous sample volume (clear) and Recovery Agent/Partitioning Oil (pink).	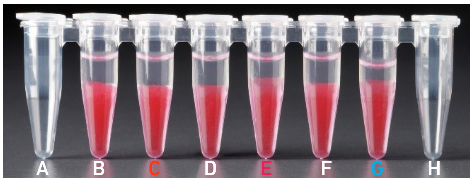<br>Tube G indicates a reagent clog has occurred. There is a decreased volume of aqueous layer (clear).<br>Tube C and E indicate a wetting failure has occurred. There is an abnormal residual volume of Recovery Agent/Partitioning Oil (pink).
2.1 b<br>After aspiration of Recovery Agent/ Partitioning Oil	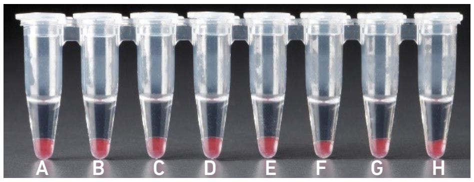<br>All liquid volumes are similar in the aqueous sample volume (clear) and residual Recovery Agent/Partitioning Oil (pink).	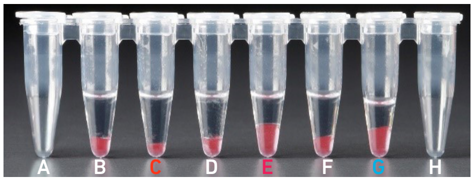<br>Tube G indicates a reagent clog has occurred. There is a decreased volume of aqueous layer (clear). There is also a greater residual volume of Recovery Agent/Partitioning Oil (pink).<br>Tube C and E indicate a wetting failure has occurred. There is an abnormal residual volume of Recovery Agent/Partitioning Oil (pink).
2.1 d<br>After addition of Dynabeads Cleanup Mix	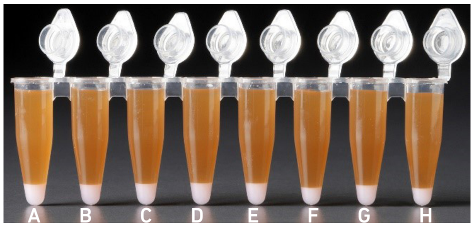<br>All liquid volumes are similar after addition of the Dynabeads Cleanup Mix.	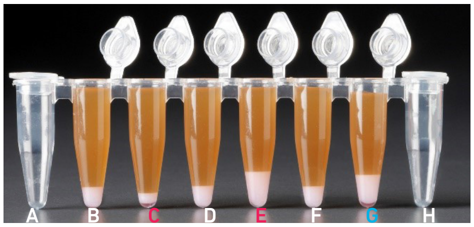<br>Tube G indicates a reagent clog has occurred. There is an abnormal ratio of Dynabeads Cleanup Mix (brown) to Recovery Agent/Partitioning Oil (appears white).<br>Tube C and E indicate a wetting failure has occurred. There is an abnormal ratio of Dynabeads Cleanup Mix (brown) to Recovery Agent/Partitioning Oil (appears white).

If a channel clogs or wetting failure occurs during GEM generation, it is recommended that the sample be remade. If any of the listed issues occur, take a picture and send it to support@10xgenomics.com for further assistance.

5.2 Chromium Controller Errors

If the Chromium Controller or the Chromium Single Cell Controller fails to start, an error tone will sound and one of the following error messages will be displayed:

a. **Chip not read – Try again:** Eject the tray, remove and/or reposition the 10x Chip Holder assembly and try again. If the error message is still received after trying this more than twice, contact support@10xgenomics.com for further assistance.

b. **Check gasket:** Eject the tray by pressing the eject button to check there is a 10x Gasket on the Chromium Chip. In the case when the 10x Gasket installation was forgotten, install and try again. In the case when a 10x Gasket was already installed, remove, reapply, and try again. If the error message is still received after trying either of these more than twice, contact support@10xgenomics.com for further assistance.

c. **Pressure not at Setpoint:**

    i. If this message is received within a few seconds of starting a run, eject the tray by pressing the eject button and check for dirt or deposits on the 10x Gasket. If dirt is observed, replace with a new 10x Gasket and try again. If the error message is still received after trying this more than twice, contact support@10xgenomics.com for further assistance.

    ii. If this message is received after a few minutes into the run, the Chromium Chip must be discarded. **Do not try running this Chromium Chip again as this may damage the Chromium Controller.**

d. **CAUTION: Chip Holder not Present:** Eject the tray by pressing the eject button to check there is a 10x Chip Holder encasing the Chromium Chip. In the case when the 10x Chip Holder was forgotten, install with a 10x Gasket in place, and try again. If the error message is still received after a 10x Chip Holder is confirmed as in place, contact support@10xgenomics.com for further assistance.

e. **Invalid Chip CRC Value:** This indicates the Chromium Chip has encountered an error, should not be run, and must be discarded. Contact support@10xgenomics.com for further assistance.

Appendix

Post Library Construction Quantification
Agilent TapeStation Traces
Oligonucleotide Sequences

6

Post Library Construction Quantification

a. Thaw KAPA Library Quantification Kit for Illumina Platforms.

b. Dilute **2 µl** sample with deionized water to appropriate dilutions that fall within the linear detection range of the KAPA Library Quantification Kit for Illumina Platforms. (For more accurate quantification, make the dilution(s) in duplicate).

c. Make enough Quantification Master Mix for the DNA dilutions per sample and the DNA Standards (plus 10% excess) using the guidance for 1 reaction volume below.

Quantification Master Mix	1X (µl)
**SYBR Fast Master Mix + Primer**	12
**Water**	4
**Total**	**16**

d. Dispense **16 µl** Quantification Master Mix for sample dilutions and DNA Standards into a 96 well PCR plate.

e. Add **4 µl** sample dilutions and **4 µl** DNA Standards to appropriate wells. Centrifuge briefly.

f. Incubate in a thermal cycler with the following protocol.

Step	Temperature	Run Time
1	95°C	00:03:00
2	95°C	00:00:05
3	67°C	00:00:30
4	Go to Step 2, 29X (Total 30 cycles)	

g. Follow the manufacturer's recommendations for qPCR-based quantification. For library quantification for sequencer clustering, determine the concentration based on insert size derived from the Bioanalyzer/TapeStation trace.

Agilent TapeStation Traces

Agilent TapeStation Traces

Agilent TapeStation High Sensitivity D5000 ScreenTape was used.
Protocol steps correspond to the Chromium Single Cell 3' Reagent Kits v3 User Guide (CG000183).

Protocol Step 2.4 – cDNA QC & Quantification

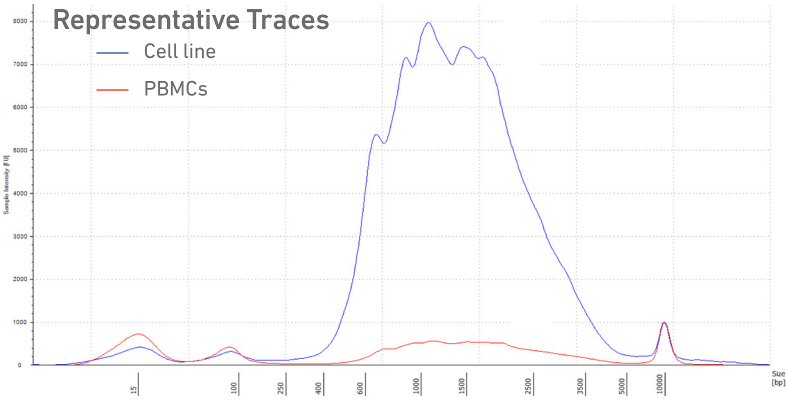

Alternate QC Method:
Qubit Fluorometer and Qubit dsDNA HS Assay Kit

Multiply the cDNA concentration reported via the Qubit Fluorometer by the elution volume (40 µl) to obtain the total cDNA yield in ng. To determine the equivalent range using the Agilent 2100 Expert Software, select the region encompassing 35-10,000 bp.

Oligonucleotide Sequences

Protocol steps correspond to the Chromium Single Cell 3' Reagent Kits v3 User Guide (CG000183)

Protocol Step 1.5 – GEM-RT Incubation

Gel Bead Primers

5'-CTACACGACGCTCTTCCGATCT-NNNNNNNNNNNNNNNN-NNNNNNNNNNNN-TTTTTTTTTTTTTTTTTTTTTTTTTTTTTTVN-3'

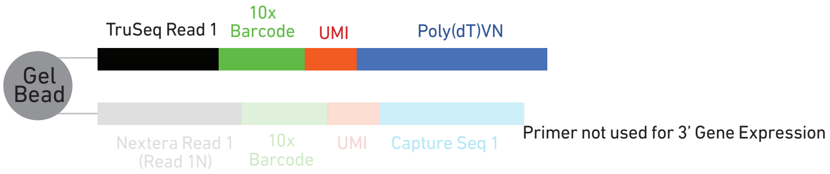

5'-GTCAGATGTGTATAAGAGACAG-NNNNNNNNNNNNNNNN-NNNNNNNNNNNN-TTGCTAGGACCGGCCTTAAAGC-3'

Template Switch Oligonucleotide PN-3000228

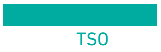

5'-AAGCAGTGGTATCAACGCAGAGTACATrGrGrG-3'

PCR Product

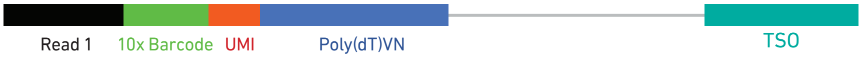

5'-CTACACGACGCTCTTCCGATCT-NNNNNNNNNNNNNNNN-NNNNNNNNNNNN-TTTTTTTTTTTTTTTTTTTTTTTTTTTTTTVN-cDNA_Insert-CCCATGTACTCTGCGTTGATACCACTGCTT-3'

Protocol Step 2.2 – cDNA Amplification

cDNA Primers PN-2000089

Forward Primer:

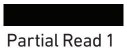

5'-CTACACGACGCTCTTCCGATCT-3'

Reverse Primer:

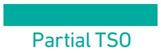

5'-AAGCAGTGGTATCAACGCAGAG-3'

Amplification Products

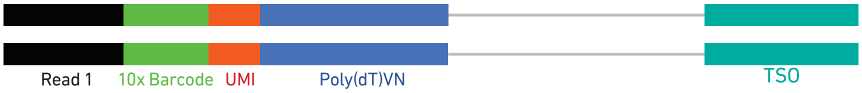

5'-CTACACGACGCTCTTCCGATCT-NNNNNNNNNNNNNNNN-NNNNNNNNNNNN-TTTTTTTTTTTTTTTTTTTTTTTTTTTTTTVN-cDNA_Insert-CCCATGTACTCTGCGTTGATACCACTGCTT-3'
3'-GATGTGCTGCGAGAAGGCTAGA-NNNNNNNNNNNNNNNN-NNNNNNNNNNNN-AAAAAAAAAAAAAAAAAAAAAAAAAAAAAABN-cDNA_Insert-GGGTACATGAGACGCAACTATGGTGACGAA-5'

Protocol Step 3.3 – Adaptor Ligation

Adaptor Oligos PN -2000094

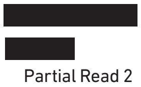

5'- GATCGGAAGAGCACACGTCTGAACTCCAGTCA-3'
3'-TCTAGCCTTCTCG-5'

Ligation Product

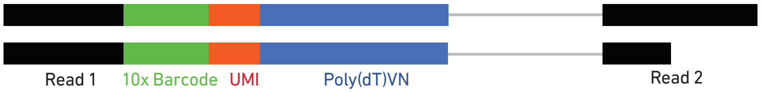

5'-CTACACGACGCTCTTCCGATCT-NNNNNNNNNNNNNNNN-NNNNNNNNNNNN-TTTTTTTTTTTTTTTTTTTTTTTTTTTTTTVN-cDNA_Insert-AGATCGGAAGAGC ACACGTCTGAACTCCAGTCAC-3'
3'-GATGTGCTGCGAGAAGGCTAGA-NNNNNNNNNNNNNNNN-NNNNNNNNNNNN-AAAAAAAAAAAAAAAAAAAAAAAAAAAAAABN-cDNA_Insert-TCTAGCCTTCTCG-5'

Oligonucleotide Sequences

Protocol Step 3.5 – Sample Index PCR

Sample Index PCR Primer PN-2000095

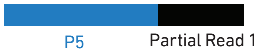

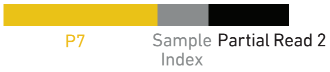

Chromium i7Sample Index PN-220103

5'-AATGATACGGCGACCACCGAGATCT-ACACTCTTTCCCTACACGACGCTC-3'

5'-CAAGCAGAAGACGGCATACGAGAT-NNNNNNNN GTGACTGGAGTTCAGACGTGT-3'

Sample Index PCR Product

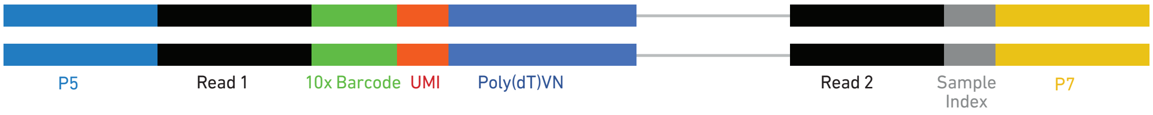

5'-AATGATACGGCGACCACCGAGATCT-ACACTCTTTCCCTACACGACGCTCTTCCGATCT-NNNNNNNNNNNNNNNN-NNNNNNNNNNNN-TTTTTTTTTTTTTTTTTTTTTTTTTTTTTTVNcDNA_InsertAGATCGGAAGAGCACACGTCTGAACTCCAGTCAC-NNNNNNNN-ATCTCGTATGCCGTCTTCTGCTTG-3'

3'-TTACTATGCCGCTGGTGGCTCTAGA-TGTGAGAAAGGGATGTGCTGCGAGAAGGCTAGA-NNNNNNNNNNNNNNNN-NNNNNNNNNNNN-AAAAAAAAAAAAAAAAAAAAAAAAAAAAAABNcDNA_InsertTCTAGCCTTCTCGTGTGCAGACTTGAGGTCAGTG-NNNNNNNN-TAGAGCATACGGCAGAAGACGAAC-5'
